# Molecular characterization of Streptococcus pyogenes (StrepA) non-invasive isolates during the 2022–2023 UK upsurge

**DOI:** 10.1099/mgen.0.001277

**Published:** 2024-08-12

**Authors:** Jennifer N. Hall, Saikou Y. Bah, Henna Khalid, Alison Brailey, Sarah Coleman, Tracey Kirk, Naveed Hussain, Mark Tovey, Roy R. Chaudhuri, Steve Davies, Lisa Tilley, Thushan de Silva, Claire E. Turner

**Affiliations:** 1 Division of Clinical Medicine, School of Medicine and Population Health, University of Sheffield, Sheffield, UK; 2 The Florey Institute of Infection, University of Sheffield, Sheffield, UK; 3 School of Biosciences, University of Sheffield, Sheffield, UK; 4 Medical Research Council Unit The Gambia at The London School of Hygiene and Tropical Medicine, Banjul, The Gambia; 5 Laboratory Medicine, Sheffield Teaching Hospitals NHS Foundation Trust, Sheffield, UK

**Keywords:** *emm*-type, group A *Streptococcus*, non-invasive, skin infection, strain diversity, *Streptococcus pyogenes*, throat infection, whole genome sequencing

## Abstract

At the end of 2022 into early 2023, the UK Health Security Agency reported unusually high levels of scarlet fever and invasive disease caused by *Streptococcus pyogenes* (StrepA or group A *Streptococcus*). During this time, we collected and genome-sequenced 341 non-invasive throat and skin *S. pyogenes* isolates identified during routine clinical diagnostic testing in Sheffield, a large UK city. We compared the data with that obtained from a similar collection of 165 isolates from 2016 to 2017. Numbers of throat-associated isolates collected peaked in early December 2022, reflecting the national scarlet fever upsurge, while skin infections peaked later in December. The most common *emm*-types in 2022–2023 were *emm*1 (28.7 %), *emm*12 (24.9 %) and *emm*22 (7.7 %) in throat and *emm*1 (22 %), *emm*12 (10 %), *emm*76 (18 %) and *emm*49 (7 %) in skin. While all *emm*1 isolates were the M1_UK_ lineage, the comparison with 2016–2017 revealed diverse lineages in other *emm*-types, including *emm*12, and emergent lineages within other types including a new acapsular *emm*75 lineage, demonstrating that the upsurge was not completely driven by a single genotype. The analysis of the capsule locus predicted that only 51 % of throat isolates would produce capsule compared with 78% of skin isolates. Ninety per cent of throat isolates were also predicted to have high NADase and streptolysin O (SLO) expression, based on the promoter sequence, compared with only 56% of skin isolates. Our study has highlighted the value in analysis of non-invasive isolates to characterize tissue tropisms, as well as changing strain diversity and emerging genomic features which may have implications for spillover into invasive disease and future *S. pyogenes* upsurges.

## Data Summary

All new genome sequence data is available on the NCBI short read archive under the bioproject PRJNA1062601, and individual accession numbers are listed in Tables S1 and S2, available in the online Supplementary Material (upplementary Materials 1 and 2).

**Impact Statement**

The human bacterial pathogen *Streptococcus pyogenes*, also known as group A *Streptococcus* or StrepA, caused a dramatic and sudden upsurge in scarlet fever in the UK at the end of 2022 into early 2023. We present molecular characterization of this upsurge, through genome sequence analysis of throat, skin and other types of non-severe infection isolates collected by the microbiology diagnostic lab at the Northern General Hospital in Sheffield, England. We found that while two strain types were the predominant cause of infections during the upsurge, other types had emerged or changed when compared with a similar collection from 2016 to 2017. We also identified differences between throat-associated isolates and skin-associated isolates and highlighted important bacterial factors that might influence infection types. Isolates from non-severe throat/skin types of infections are rarely saved, and, therefore, our knowledge of them is limited. However, here, we demonstrate that the study of such isolates may be key to understanding upsurges of more severe infections.

## Introduction

The human pathogen *Streptococcus pyogenes*, also known as group A *Streptococcus* or StrepA, is a common cause of throat infections, such as pharyngitis and tonsillitis, as well as mild skin infections such as pyoderma or impetigo. More commonly in children than adults, throat infections can progress to scarlet fever, with a characteristic sandpaper-like rash and ‘strawberry tongue’. On rare occasions, *S. pyogenes* can also cause severe and potentially lethal invasive diseases, such as pneumonia, empyema, bacteraemia and necrotizing fasciitis (‘flesh-eating’ disease). In England, scarlet fever and invasive *S. pyogenes/*group A *Streptococcus* (iGAS) disease cases are notifiable to the UK Health Security Agency (UKHSA).

In September 2022, the UKHSA reported an unusually high level of scarlet fever notifications with ~3.7-fold more than in the same period for the previous five seasons [1]. Notifications continued to rapidly increase across England and Wales, with 8688 cases in weeks 37–48 (mid-September to end-November), compared with 333–2536 in the previous five seasons [2]. Alongside the increasing scarlet fever cases, there were also high numbers of iGAS notifications with 772 in weeks 37–48. Concerningly, during these weeks, there were more cases of iGAS in children under 15 (26.1%), compared with previous seasons (6.4–13.3%), and 14 deaths in this age group [2]. Scarlet fever notifications peaked in week 49 with 10 069 cases, and iGAS notifications peaked in week 52 with 213 cases [3]. Typing of isolates by the sequence of the *emm* gene, which encodes for the hypervariable M protein, identified *emm*1 as the most common cause of iGAS in those older than 15 (31 %) but an even higher proportion of cases in those younger than 15 (57 %). *emm*12 and *emm*4 were the second (23 %) and third (7 %) most common causes of iGAS in children, at least in the early part of the upsurge [2].

Scarlet fever demonstrates seasonality with cases typically increasing in late winter and peaking in early spring. An unexpected increase in scarlet fever cases in England was first seen in 2013–2014, peaking in early April and totalling over 13 000 notifications, compared with fewer than 3000 in previous seasons [4,5]. Notifications remained seasonally elevated, rising each year to their highest in 2017–2018 [6], until the COVID-19 (coronovirus disease 2019) pandemic when cases fell dramatically. No corresponding rise was observed for iGAS notifications until the 2015–2016 season. The link between scarlet fever cases and iGAS cases is not well understood, but it appeared that the increase in both scarlet fever and invasive disease notifications in early 2016 was due to the emergence of a new variant of a lineage carrying *emm*1 termed M1_UK_ [7,8], which, prior to 2022–2023, also led to the biggest upsurge in cases in 2017–2018 [6]. The M1_UK_ variant had steadily increased in prevalence in England since 2010 and represented 91.5 % of invasive *emm*1 by 2020 [8]. M1_UK_ is characterized by 27 SNPs in comparison to a globally circulating *emm*1 population (M1_global_) and increased expression of the superantigen SpeA [7,9, 10]. The M1_UK_ lineage has been detected in European countries, North America, Australia and New Zealand, often associated with increases in disease [7,9, 11,17].

While iGAS undergoes routine surveillance in several high-income countries, with the inclusion of scarlet fever and outbreak situations in some, these types of infections only represent a small proportion of streptococcal cases. Our knowledge of circulating non-invasive disease (from non-sterile sites) isolates is severely lacking, yet these isolates may act as an early indicator for increasing prevalence of new genotypes or lineages that could lead to more severe infections. This lack of knowledge also means we have a limited understanding of the connection between certain *emm*-types or lineages and preferences for causing throat infections or skin infections. As non-invasive isolates are not routinely collected, data acquisition relies on local collections.

Sheffield is a large northern city in England with a population of around 600 000. During the 2022–2023 upsurge, the region of Yorkshire and the Humber, which includes Sheffield, reported the highest rates of invasive *S. pyogenes* in England, at 8.7 per 100 000 population [3]. Scarlet fever notifications were also high in the region, at 132.0 per 100 000 population, although this was similar to the rate seen in the North West region and lower than the East Midlands region [3]. We began in November 2022 to routinely save all non-invasive isolates identified by the Department of Laboratory Medicine at the Northern General Hospital, Sheffield, which performs microbiological services for community care as well as surrounding hospitals. We had also previously performed a similar collection in 2016–2017 which we used as a comparative population. We undertook whole genome sequencing (WGS) analysis of both collections and characterized strain diversity and pathogenicity factors. As expected, the *emm*1 M1_UK_ lineage dominated during the 2022–2023 upsurge followed by *emm*12, but there were some unexpected increases in other *emm*-types as well as differences between throat-associated isolates and skin-associated isolates.

## Methods

### Isolate collection

A total of 384 non-invasive isolates (from non-sterile sites), presumptively identified from culture as *S. pyogenes*, were collected from the Department of Laboratory Medicine, Northern General Hospital, Sheffield, UK, between November 2022 (week 45) and February 2023 (week 6). The Department of Laboratory Medicine performs microbiology diagnostics for NHS Trusts as well as primary care and community services, acting as the single regional diagnostic microbiology laboratory for a population of around 600 000 adults and children in Sheffield. Anonymized clinical data were collected for each isolate from the sample request information and electronic clinical patient records: swab source (throat/skin/ear/eye/nose), sampling date, age, sex and infection type. Cases were considered to be associated with scarlet fever where scarlet fever was queried by the clinician or a rash consistent with a scarlet fever diagnosis was described in the clinical details accompanying the request. All other throat samples, and other sample types, were deemed non-scarlet fever samples. Samples from ear, eye and nose isolates were excluded from further downstream analyses to focus the comparison on throat and skin isolates.

For comparison, a further 229 archived non-invasive *S. pyogenes* isolates collected in a similar manner between October 2016 and January 2017 were also included in this study, with data collected as above. The 2016–2017 season also showed a seasonal upsurge in disease compared with previous years, however with substantially fewer scarlet fever and invasive *S. pyogenes* cases reported compared with 2022–2023. The age distribution of cases in 2016–2017, of both scarlet fever and invasive *S. pyogenes* disease, was consistent with previous years, and this was therefore considered a suitably representative comparative sample.

### Whole genome sequencing

Genomic DNA was extracted from all isolates using a previously described method [18]. Genomic DNA from isolates collected in 2022–2023 underwent WGS at Earlham Institute by the Genomics Pipeline group, using the LITE protocol [19] for library preparation and sequenced on a NovaSeq X plus generating 150 bp paired-end reads. Genomic DNA from isolates collected in 2016 underwent sequencing provided by MicrobesNG (https://microbesng.com) using the Nextera XT library prep kit (Illumina) and the Illumina HiSeq 2500, generating 250 bp paired-end reads.

### WGS analysis

Raw sequence reads were trimmed using Trimmomatic (v0.39) with the settings LEADING:3 TRAILING:3 SLIDING WINDOW:4 : 15 MINLEN:36 [20]. For the 2022–2023 isolates, the average estimated read coverage was ~524× with some greater than 1000×. Reads were therefore randomly subsampled, using seqtk, to 1.2 million reads per isolate, to provide ~180× coverage.

Trimmed and subsampled reads were then used to perform *de novo* assembly using SPAdes (v.3.13.1) with k-mer sizes of 21, 33, 55 and 77 [21]. Assembly statistics were generated for each isolate using Quast [22] (Tables S1 and S2), and any draft assemblies with more than 500 contigs or a total genome size greater than 2.2 Mb were excluded from downstream analysis, as were any that were determined were not to be *S. pyogenes* based on their genomic sequences. MLST and *emm*-types were determined from the *de novo* assemblies using mlst (https://github.com/tseemann/mlst) with the pubmlst database [23] and the emm_typer.pl script (github.com/BenJamesMetcalf/GAS_Scripts_Reference), respectively. New MLSTs were submitted to pubmlst and new *emm*-types and sub-types to the CDC *emm*-type database (https://cdc.gov/streplab) for assignment.

*De novo* assemblies were annotated using Prokka (v.1.14.6) [24]. Snippy (https://github.com/tseemann/snippy) was used to determine SNP distances between sequence reads and a reference genome. RAxML v8.2.12 [25] was used to generate maximum likelihood phylogenetic trees based on the core gene alignment with a general time-reversible substitution model and 100 bootstraps. Known regions of recombination (prophage regions and other mobile genetic elements) were excluded from reference genomes prior to mapping. Where these regions were unknown in the *emm*22 reference genome, regions of predicted recombination were identified and removed using Gubbins [26] prior to tree construction. Phylogenetic trees were annotated using iTOL (version 6) [27].

Further comparison was made with previously published WGS data from Sheffield, consisting of 142 non-invasive skin and soft tissue isolates collected in 2019 [28], and national and international data from other published collections (Table S3).

### Virulence factor, regulator and antimicrobial resistance typing

The presence of superantigen genes *speA*, *speC*, *speG*, *speH*, *speI*, *speJ*, *speK*, *speL*, *speM*, *speK/M*, *speQ*, *speR*, *ssa* and *smeZ* and DNase genes *sda1*, *sda2*, *sdn*, *spd1*, *spd3* and *spd4* was determined by blast (100 % coverage, 80 % identity) with representative gene sequences against assemblies and a manual check where needed. For other genes (*covR/S*, *rocA*, *hasABC* and the *nga-ifs-slo* locus with promoter region), sequences were extracted from the assembled genomes and compared with the reference genome, H293 (GenBank accession NZ_HG316453.1), as previously described [28]. Antimicrobial resistance gene carriage was determined with ABRicate (https://github .com/tseemann/abricate) using the NCBI database [29].

### Statistical analysis

Statistics were performed in GraphPad Prism (v10.2.3).

## Results

### Collected isolates

During the unusual *S. pyogenes* infection upsurge period reported by the UKHSA in England in 2022–2023, we collected a total of 384 non-invasive isolates from the Department of Laboratory Medicine, Sheffield. This collection was compared with 229 isolates collected in 2016–2017, representing a time of more typical seasonal upsurge. The proportion of throat isolates was similar for both time periods (61.7% and 65.1 %), but during 2022–2023, overall isolate numbers were much higher (Table 1). Significantly more throat isolates were also associated with scarlet fever in 2022–2023 than in 2016–2017 [24.5 % vs 5.4 %, *χ*^2^(1) = 23.55, *P*<0.0001]. For both time periods, throat isolates were primarily from children under 9, but in 2022–2023, significantly more were from children aged 5–9 compared with 2016–2017 [42.6 % vs 19.5 %, *χ*^2^(1) = 21.96, *P*<0.0001]. There were fewer skin than throat isolates for both time periods, and skin isolates were predominantly from those aged 1 year and under and those aged 45 and older. There was evidence in both time periods of a bimodal age distribution with the highest number of isolates from children under 9 years of age and a second peak in individuals aged 30–39 years (Fig. S1). *S. pyogenes* was more frequently isolated from throat swabs in female patients [57.6 %(136/236) in 2022–2023 and 63.8 % (95/149) in 2016–2017] and from skin swabs in male patients [68.8 % (77/112) in 2022–2023 and 63.2 % (43/68) in 2016–2017] (Fig. S1).

**Table 1.** Clinical characteristics of isolates collected in 2022–2023 and 2016–2017

	2022–2023 (%)	2016–2017 (%)
Clinical presentation		
Throat	237 (61.7)	149 (65.1)
Scarlet fever	58 (24.5)	8 (5.4)
No scarlet fever	179 (75.5)	141 (94.6)
Skin	111 (28.9)	68 (29.7)
Others	36 (9.4)	12 (5.2)
Ear	28 (7.3)	10 (4.4)
Eye	7 (1.8)	0 (0)
Nose	1 (0.3)	2 (0.9)
Sex		
Female	190 (49.5)	125 (54.6)
Male	194 (50.5)	104 (45.4)
Age*		
1 year and under	26 (6.8)	10 (4.4)
2–4 years	64 (16.7)	38 (16.6)
5–9 years	128 (33.3)	36 (15.7)
10–14 years	33 (8.6)	20 (8.7)
15–44 years	97 (25.3)	103 (45.0)
45–64 years	22 (5.7)	13 (5.7)
65–74 years	9 (2.3)	5 (2.2)
75 years and over	5 (1.3)	4 (1.7)
Total	384 (100)	229 (100)

#*Isolates are grouped in accordance with UKHSA age categories.

### *emm*-type distribution

After quality control filtering, a total of 341 whole genome sequences from the *S. pyogenes* isolates collected in 2022–2023 were included in study analyses: 209 (61.3 %) from throat swabs, 54 (25.8 %) of which were associated with scarlet fever, 100 (29.3 %) from skin swabs and 32 (9.4 %) from other sites. From the 2016–2017 collection, 165 isolates passed quality control filtering: 127 (77.0 %) from throat swabs and 38 (23.0 %) from skin swabs. We purposely did not sequence isolates from ‘other’ sites in this earlier collection.

**Fig. 1.**
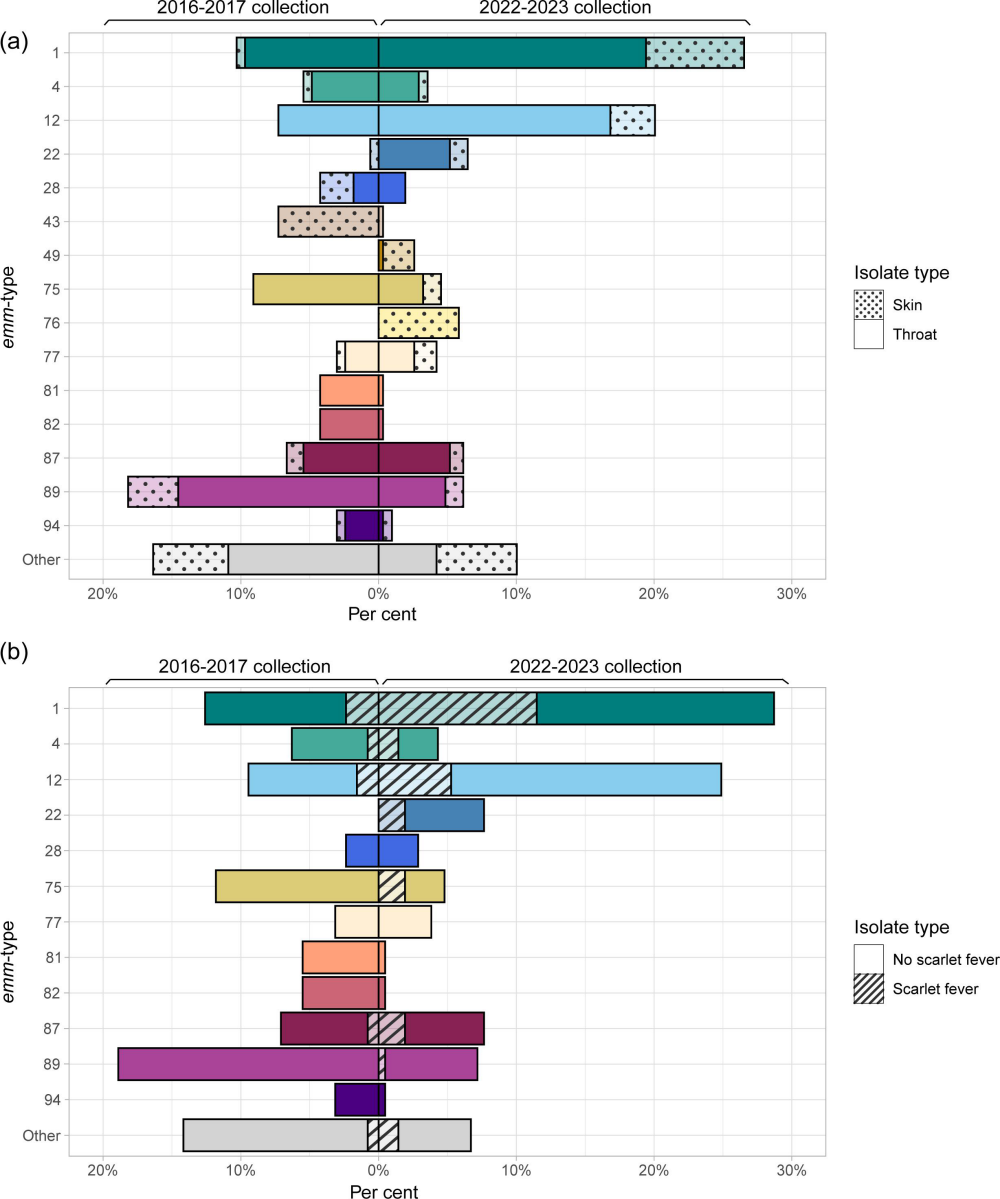
(**a**) Distribution of *emm*-types by collection year and clinical isolate type. (**b**) Distribution of *emm*-types within throat isolates by clinical presentation. Data presented as a percentage of (**a**) the total number of isolates (*n*=341 for 2022–2023, *n*=165 for 2016–2017) and (**b**) the total number of throat isolates (*n*=209 for 2022–2023, *n*=127 for 2016–2017) in each collection period.

**Fig. 2.**
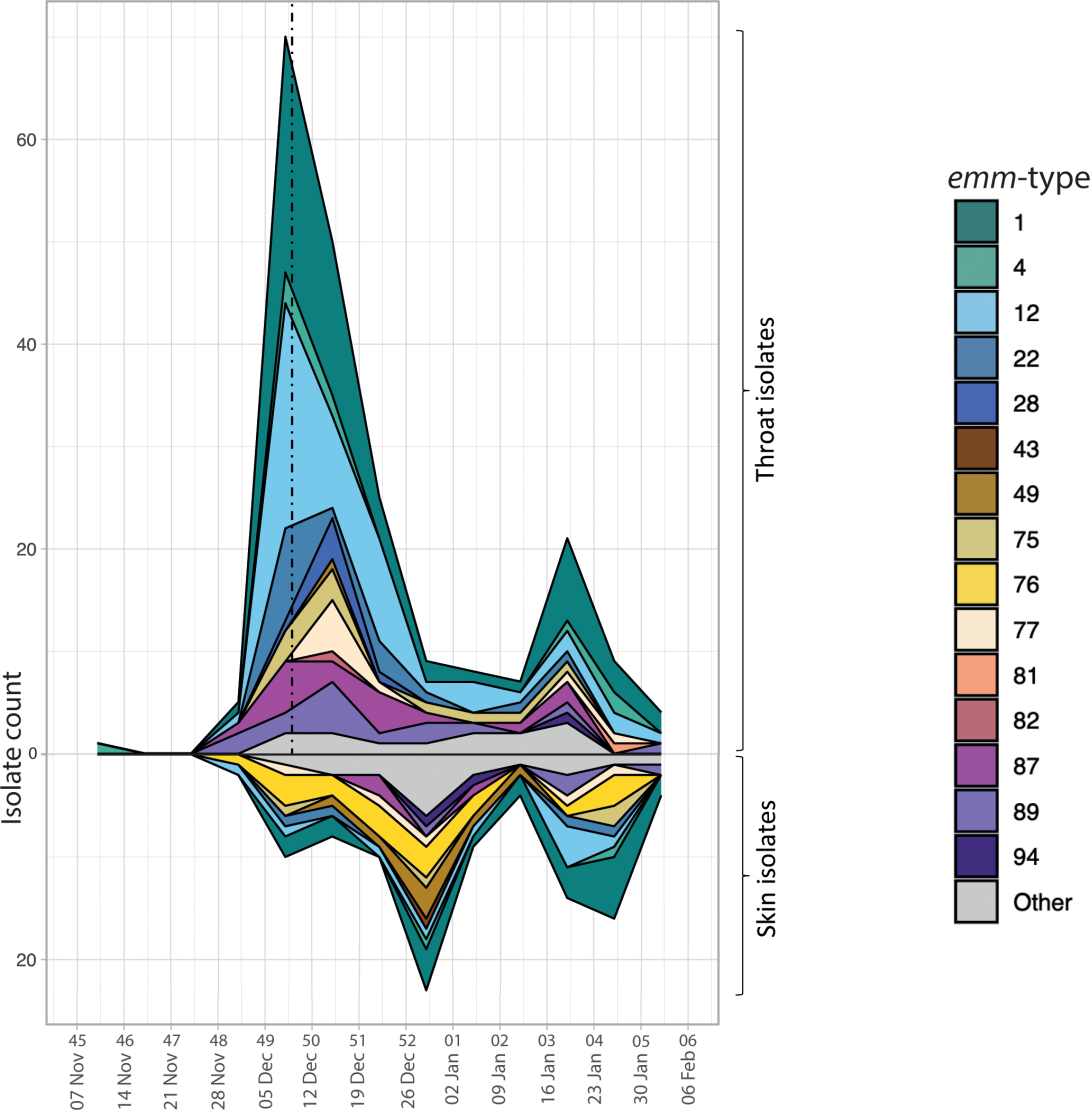
Distribution of *emm*-types across time for throat and skin isolates collected in 2022–2023. Week numbers and dates are presented on the x-axis. The dashed line represents the introduction of the NHS England group A *Streptococcus* interim clinical guidance summary for case management, on the 9^th^ of December 2022.

The *emm*-type for each isolate was extracted from the WGS data. The frequency of isolates and the distribution of *emm*-types varied over time in the 2022–2023 collection, with the total number peaking in week 49, in keeping with UKHSA data [3]. We observed a fall in the number of throat swabs following the issue of interim clinical guidance by NHS England on the 9th of December 2022 (week 49); this guidance advised clinicians to lower their threshold to empirically treat children with sore throats, including when their presentation may be secondary to viral respiratory illness. Across all 2022–2023 isolates, a total of 33 different *emm*-types were identified, with *emm*1 being the most common at 95/341 (27.9 %) followed by *emm*12, at 64/341 (18.8 %) (Fig. 1a). This high level of *emm*1 and *emm*12 cases was reflected in an increase in the number of throat samples in late November to early December 2022 (Fig. 2). Within our comparative collection from 2016–2017, 28 different *emm*-types were identified overall, most frequently *emm*89 (30/165, 18.2 %).

Within the 2022–2023 throat isolates, 22 different *emm*-types were identified, but just *6 emm*-types made up 80.9 % of isolates: *emm*1 (28.7 %, 60/209), *emm*12 (24.9 %, 52/209), *emm*22 (7.7 %, 16/209), *emm*87 (7.7 %, 16/209), *emm*89 (7.2 %, 15/209) and *emm*75 (4.8 %, 10/209). A significantly higher proportion of throat isolates were *emm*1 and *emm*12 in 2022–2023 compared with 2016–2017, where 12.6 % (16/127) isolates were *emm*1 [*χ*^2^(1) = 11.71, *P*=0.0006] and 9.5 % (12/127) were *emm*12 [*χ*^2^(1) = 12.20, *P*=0.0005]. In the 2022–2023 throat isolates, *emm*89 made up a significantly lower proportion than the 2016–2017 collection, in which it was the most common *emm*-type at 18.9 % (24/127) isolates [*χ*^2^(1) = 10.58, *P*=0.0011]. There was also a lower proportion of *emm*75 within the 2022–2023 throat isolates compared with 2016–2017, where *emm*75 made up 11.8 % (15/127). *emm*87 made up a similar proportion in 2016–2017 (7.1 %, 9/127); however, no throat isolates were *emm*22 in 2016–2017. Throat isolates associated with scarlet fever in 2022–2023 were predominantly *emm*1 (44.4 %, 24/54) and *emm*12 (20.4 %, 11/54) (Fig. 1b). In 2016–2017, scarlet fever was associated with 8/127 throat isolates of five *emm*-types: *emm*1 (3/8), *emm*12 (2/8), *emm*4 (1/8), *emm*6 (1/8) and *emm*87 (1/8).

The 2022 upsurge in throat disease was followed by a surge in skin disease in late December 2022, with a second peak in late January 2023 (Fig. 2). This was driven by 23 different *emm*-types, most frequently *emm*1 (22/100, 22 %) and *emm*12 (10/100, 10 %), but also *emm*76 (18/100, 18 %) and *emm*49 (7/100,7 %), both of which were rarely found in the throat. This differed from our 2016–2017 collection, for which there were 17 different *emm*-types but *emm*43 (31.6 %, 12/38) was dominant, followed by *emm*89 (15.8 %, 6/38) and *emm*28 (10.5 %, 4/38) (Fig. 1a). Only a single skin isolate was *emm*1, and none were *emm*12 or *emm*76.

**Fig. 3.**
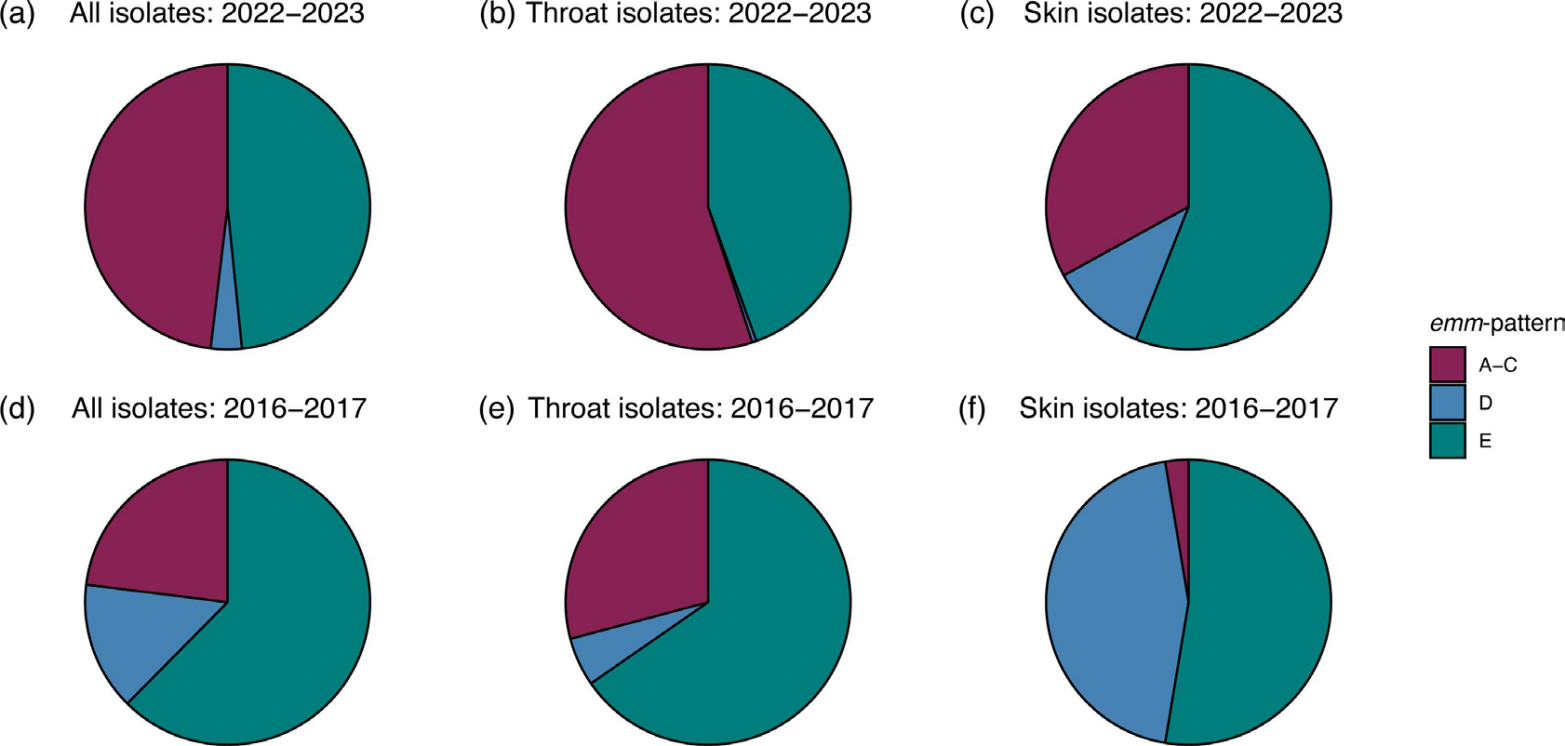
Distribution of *emm*-patterns across 2022–2023 and 2016–2017 collections. (**a**) all 2022–2023 isolates (*n*=341); (**b**) 2022–2023 throat isolates (*n*=209); (**c**) 2022–2023 skin isolates (*n*=100); (**d**) all 2016–2017 isolates (*n*=165); (**e**) 2016–2017 throat isolates (*n*=127); (**f**) 2016–2017 skin isolates (*n*=38). Pie charts represent the percentage of isolates associated with each pattern.

An *emm*-pattern was assigned to each isolate using previous *emm*-type assigned patterns based on the genes surrounding the *emm*-gene [30]. Within the 2022–2023 collection, there was a similar proportion of ‘throat-associated’ *emm*-pattern A–C (48.1 %, 164/341) and ‘generalist’ *emm*-pattern E (48.4 % 165/341), with just 3.5 %(12/341) being ‘skin-associated’ pattern D (Fig. 3). All pattern D isolates were of skin origin, except a single throat isolate. Within the 2016–2017 collection, ‘generalist’ *emm*-pattern E was more common at 62.4 % (103/165); 23 % (38/165) were patterns A–C. With 14.5 % (24/165) of isolates pattern D, the 2016–2017 collection had a greater proportion of pattern D than the 2022–2023 collection overall, with the most striking difference noted in the skin isolates, where pattern D made up 44.7 %(17/38) of the 2016–2017 skin isolates but just 11 % (11/100) of the 2022–2023 skin isolates. Just one skin isolate collected in 2016–2017 was of *emm*-pattern A–C. Despite these differences and consistent with their previously defined tissue tropism, in both time periods, *emm*-pattern A–C were significantly more associated with throat isolates than skin [2016–2017 *χ*^2^(1) = 11.59, *P*=0.0007; 2022–2023 *χ*^2^(1) = 13.15, *P*=0.0003], while *emm*-pattern D was significantly more associated with skin isolates than throat [2016–2017 *χ*^2^(1) = 36.20, *P*<0.0001; 2022–2023 *χ*^2^(1) = 20.06, *P*<0.0001], and pattern E isolates were not significantly associated with one or the other.

Each isolate was also assigned an *emm*-cluster, a functional classification grouping closely related M proteins that share binding and structural properties (Fig. S2). Within the 2022–2023 collection, the most common was the *emm*1 cluster type A-C3 (27.9 %, 95/341) and the *emm*12 cluster type A-C4 (18.8 %, 64/341). E4 was also common (19.6 %, 67/341), representing *emm*22, *emm*77, *emm*89 and *emm*102. By comparison, *emm-*cluster E4 made up the largest proportion of isolates within the 2016–2017 collection (28.5 %, 47/165), reflecting the high number of *emm*89 isolates from both throat and skin samples. The 2016–2017 collection also had a number of samples in cluster D4 (9.7 %, 15/165), all of which were skin isolates and mostly *emm*43 (80 %, 12/15).

### Antimicrobial resistance genes

The presence of antimicrobial resistance genes in our 2022–2023 throat and skin isolate genomes was relatively low overall (Table S1), with only 29.4 % (91/309) carrying at least one gene. By far the most common was *tetM* at 24.3 % (75/309) and 28.5 % of isolates carried at least one *tet* gene (*tetL*, *tetM*, *tetO* or *tetT*). The second most common resistance gene was *ermA* (4.9 %). No resistance genes were identified in *emm*1, and only three *emm*12 carried resistance genes (*mefA* and *msrD* encoding macrolide resistance). Fewer 2016–2017 throat and skin isolate genomes carried at least one resistance gene (18.2 %, 30/165) (Table S2), but all of these included a *tet* gene (*tetM* or *tetO*), with *tetM* being the most common at 15.8 % (26/165).

### Hyaluronic acid capsule synthesis and the *nga-ifs-slo* toxin loci

We previously identified an increasing number of *emm*-types that had undergone recent genetic changes leading to the inability to produce the hyaluronic acid capsule, through loss or nonsense mutation of capsule synthesis genes *hasABC* [31]. Forty per cent of our Sheffield 2022–2023 throat and skin isolates were predicted to be unable to synthesize the hyaluronic acid capsule due to nonsense mutations in or the absence of the *hasA*, *hasB* and *hasC* genes. While sporadic nonsense mutations occurred in *hasA* or *hasB* in some *emm*-types, such as *emm*1 (4.9 %) and *emm*12 (14.5 %), 100 % of all isolates belonging to 11 different *emm*-types were predicted to be acapsular (Table S1). This included *emm*22 and *emm*89, for which, as expected, the entire *hasABC* locus was absent, *emm*28, *emm*77 and *emm*87 which had previously described nonsense mutations in *hasA* [31,32], and all *emm*9, *emm*29, *emm*58, *emm*81, *emm*90 and *emm*94 isolates. Typically, *emm*4 also lacked the *hasABC* locus although one isolate was found to carry it. Nonsense *hasA* mutations were also found in the majority of *emm*75 (78.5 %) and *emm*11 (66.7 %).

Only 51 % of 2022–2023 throat isolates were predicted to be able to produce capsule compared with 78 % of 2022–2023 skin isolates. Loss of capsule was predominantly associated with *emm*-pattern E isolates, for which only 27 % were predicted to be encapsulated compared with 91 and 92 % of patterns A–C and D, respectively. This was similar in the 2016–2017 isolates, where only 30 % of pattern E isolates were predicted to be able to make capsule (Table S2).

We previously identified convergent evolution with acapsular isolates also having undergone homologous recombination resulting in increased expression of the toxins NADase and streptolysin O (SLO) [31,33, 34]. High or low expression of these toxins can be linked to three residues in the promoter region of the *nga* (encoding for NADase), *ifs* (encoding the inhibitor of NADase) and *slo* locus [34]. Within the 2022–2023 throat isolates, 90 % were predicted to have high-toxin expression (as defined previously [31]), compared with 56 % of skin isolates (Table S1). Only 8 % of pattern D isolates were predicted to have high-toxin expression, compared with 99 % of A–C and 66 % of E. Additionally, only 58 % of pattern D isolates would express active NADase, based on a glycine residue at codon 330 rather than an aspartate [35], compared with 99% and 100 % of pattern A–C and E isolates, respectively. Although *emm*1 and *emm*12 isolates were predominantly encapsulated with a high-toxin expression genotype, an acapsular with a high-toxin expression genotype was found in 41 % of throat isolates compared with a significantly lower 22 % of skin isolates [*χ*^2^(1) = 10.91, *P*=0.001]. Only 2 % of throat isolates were predicted to be encapsulated with low-toxin expression genotype compared with 44 % of skin isolates. This was similar in 2016–2017 with 8 % of throat isolates predicted to be encapsulated with a low-toxin expression genotype but 50 % of skin isolates (Table S2). Interestingly, the association of capsule and toxin expression genotype with infection site was maintained even within pattern E isolates. A significantly higher proportion of 2022–2023 pattern E isolates from the skin were encapsulated with a low toxin expression genotype compared with the pattern E throat isolates [58.9 % vs 3.2 %, *χ*^2^(1) = 59.19, *P*<0.0001] and the same within 2016–2017 pattern E isolates [52.6 % vs 7.9 %, *χ*^2^(1) = 39.39, *P*<0.0001]. Conversely, a significantly higher proportion of 2022–2023 pattern E isolates from the throat were acapsular with a high-toxin expression genotype compared with pattern E skin isolates [78.5 % vs 37.5 %, *χ*^2^(1) = 25.22, *P*<0.0001], and a higher but not significantly different proportion of 2016–2017 pattern E throat isolates compared with skin isolates [47.2 % vs 31.5 %, *χ*^2^(1) = 2.918, *P*=0.088].

### Superantigens

*S. pyogenes* has the potential to carry at least one of 13 different superantigen genes: *speG*, *speJ*, *speQ*, *speR* and *smeZ* are chromosomal, while *speA*, *speC*, *speH*, *speI*, *speK*, *speL*, *speM* and *ssa* are prophage-associated. Within 2022–2023 throat and skin isolates, 96.1 % (297/309) carried *smeZ* and 92.2 % (285/309) carried *speG* (Fig. S3A). The other chromosomal superantigens were seen less frequently, with *speJ* in 42.4 % (131/309) and the co-transcribed *speQ* and *speR* in 12.6 % (39/309). Of the prophage-associated superantigens, *speC* was the most common at 52.4 % (162/309), followed by *speA* at 32 % (99/309). One *emm*49 skin isolate carried a *speK/speM* fusion gene which we previously identified in an *emm*65 [28]. Of the 99 isolates that carried *speA*, 80.8 % (80/99) were *emm*1.

In comparison, a similar proportion of isolates from the 2016–2017 collection carried the most common superantigen genes, *smeZ* and *speG*, at 94.5 % (156/165) and 90.9 % (150/165), respectively (Fig. S3B). Prophage-associated *speC* was found in 64.8 % (107/165) of isolates in 2016–2017, slightly higher than in 2022–2023. However, the number of isolates carrying *speJ* and *speA* was lower in 2016–2017, where *speJ* was found in 22.4 % (37/165) and *speA* in 16.4 % (27/165); this difference reflects the 97.6 % (80/82) of 2022–2023 *emm*1 isolates carrying this combination of superantigens. In 2022–2023, 22 % (68/309) carried *speI* compared with 16.4 %(27/165) in 2016–2017, reflecting the upsurge in *emm*12 in 2022–2023, all of which carried *speI*. A lower proportion had *speK* in 2022–2023 (14.6 %, 45/309) than in 2016–2017 (26.1 %, 43/165), reflecting the single *emm*43 isolate in 2022–2023 compared with 12 *emm*43 skin isolates in 2016–2017. One *emm*25 skin isolate in 2016–2017 carried a *speK/speM* fusion gene.

### DNases

All *S. pyogenes* isolates carry at least one DNase gene, out of a possible two chromosomal and six prophage-encoded genes [36]. Within the 2022–2023 throat and skin isolates, the most common prophage-associated DNase genes identified were *spd3* in 60.5 % (187/309), *spd1* (carried with *speC*) in 52.4 % (162/309) and *sda2* in 43.0 % (133/309) (Fig. S4A). Most DNase combinations were seen in a range of *emm*-types, although the most common combinations were specific to *emm*1 (*sda2* with *spd3*, 23.6 %, 73/309) and *emm*12 (*sda2* with *spd1,* 13.3 %, 41/309). By comparison, in the 2016–2017 collection, *sda2* was seen in fewer isolates, at 21.2 % (35/165), and *spd1* in slightly more, at 64.2 % (106/165), but *spd3* in similar numbers, at 56.4 % (93/165) (Fig. S4B). In both collections, *sdn* was less common, and *spd4* was quite rare, with *spd4* only being present with *spd3* in a single isolate in 2022–2023 and two isolates in 2016–2017 (two *emm*5 and one *emm*87); all other isolates carrying *spd3* or *spd4* had just one of these two DNase genes.

### CovR, CovS and RocA regulator proteins

The two-component regulator CovR/S and the regulator of Cov, RocA, negatively regulate key *S. pyogenes* virulence factors including capsule and toxins. For both collections, nonsense or frameshift mutations leading to premature stop codons that would alleviate virulence factor repression were rare in CovS and RocA and absent in CovR. Only one 2022–2023 isolate had a premature stop codon in CovS (an *emm*1) and two 2016–2017 isolates (one *emm*1 and one *emm*89). A total of 2.6 %(8/309) 2022–2023 throat or skin isolates carried premature stop codons that would truncate RocA. Consistent with previous findings, all *emm*3 isolates from both collections would express a truncated RocA after 416 aa. No other *emm*-types had nonsense mutations in *rocA* in 2016–2017. Other non-synonymous variations were found in *covS* and *rocA* (Tables S1 and S2), but the impact of these is difficult to determine, and many were associated with *emm*-type.

Although nonsense mutations in *covR* were absent, other non-synonymous variations in *covR* were found in 45/309 (14.6 %) of 2022–2023 skin and throat isolates. However, the majority were related to the *emm*-type. All *emm*22 isolates from 2022–2023 (*n*=20) and 2016–2017 (*n*=1) had the same variation in CovR (V128A) and an additional variation in RocA (V333A). Nearly all *emm*77 isolates from both 2022–2023 (12/13) and 2016–2017 (4/5) had the same M170I variation in CovR. 14.5 % of 2022–2023 *emm*12 isolates (9/62) had an A105G variation in CovR. Four other isolates of different *emm*-types had other CovR variations.

### *emm*1

**Fig. 4.**
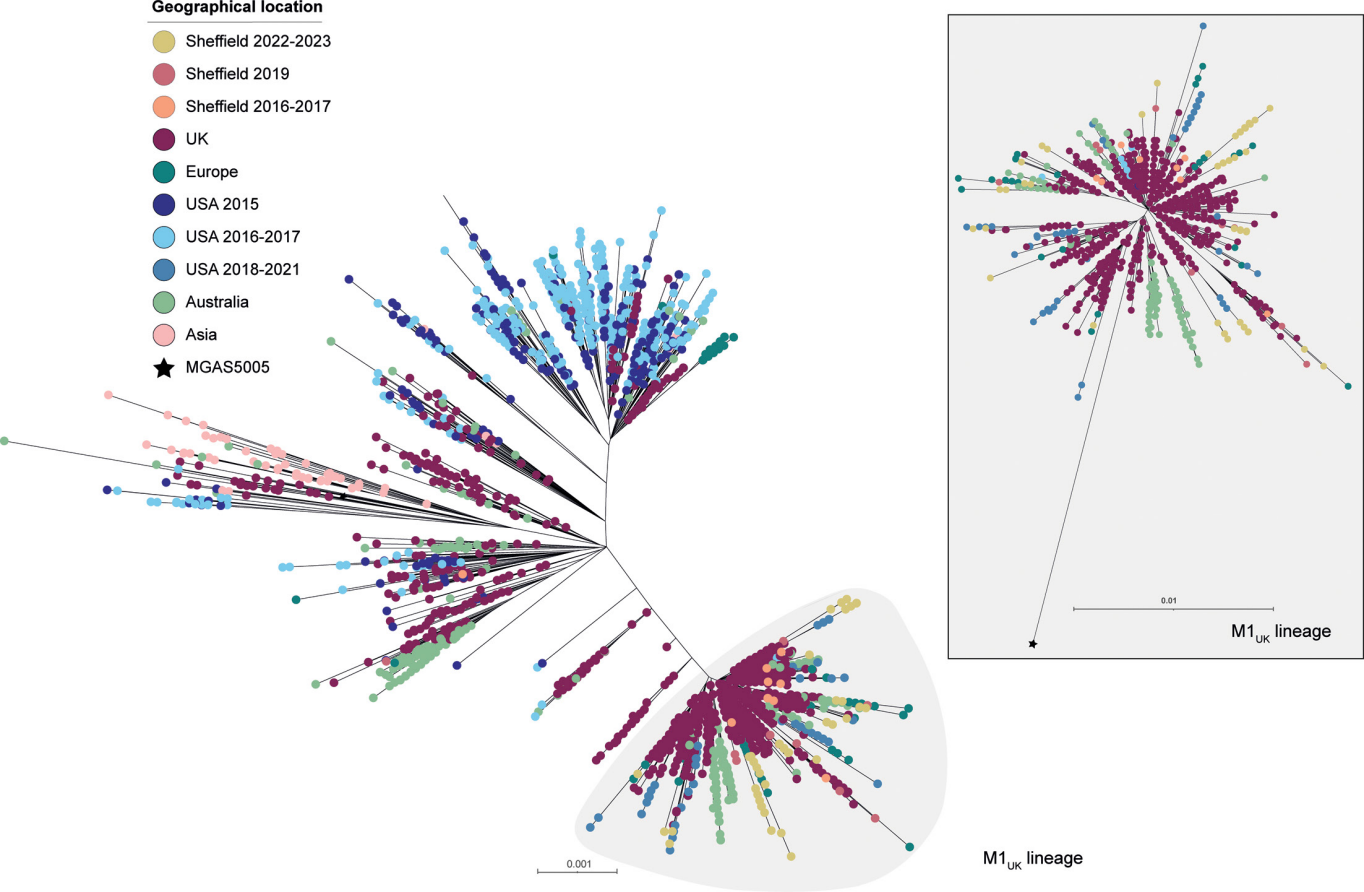
Phylogenetic analysis of Sheffield *emm*1 genomes collected in 2022–2023 and 2016–2017, within the context of global *emm*1 isolates. A maximum likelihood phylogenetic tree was generated with SNPs extracted from a core gene alignment (excluding prophage and SpyCI regions) to reference *emm*1 strain MGAS5005 [54]. Alongside our Sheffield *emm*1 genomes from 2022–2023 and 2016–2017, we included publicly available *emm*1 genome data from Sheffield, 2019 (*n*=14) [28]; other UK sites, 2001–2018 (*n*=1399) [7,31, 55,59]; Denmark, 2018–2023 (*n*=98) [14]; Netherlands, 2019 (*n*=15) [13]; Portugal, 2022–2023 (*n*=30) [16]; Australia, 2005–2020 (*n*=318) [9]; China, 2004–2016 (*n*=64) [60,61]; the USA, 2015 (*n*=316) [38], 2016–2017 (*n*=468) [62] and 2018–2021 (*n*=85) [11]. The scale bar represents the number of nucleotide substitutions per site. The grey shaded area indicates the emergent lineage M1_UK_ defined by 27 SNPs; this lineage is shown as a separate tree within the panel.

A total of 95 (27.9 %) of the 341 genomes collected in 2022–2023 were *emm*1. Sixty (63.2 %) of these were from throat isolates, of which 24 were associated with scarlet fever. Twenty-two (23.2 %) were skin isolates, and the remaining 13 were from other sites including 12 from ear swabs and one from an eye swab. All these *emm*1 isolate genomes clustered within the M1_UK_ lineage, and all carried the 27 lineage defining SNPs (Fig. 4). Similarly, for our 2016–2017 *emm*1 isolates, only one out of 17 *emm*1 isolates was not M1_UK_. This is in keeping with the emergence of M1_UK_ as the dominant *emm*1 strain globally. Sheffield isolates were spread throughout the M1_UK_ phylogeny without evidence of expansion of a specific sub-clade. Two scarlet fever throat isolates lacked the *speA*-carrying Φ5005.1 phage. All other isolate genomes had *speA*; the chromosomal *speG*, *speJ* and *smeZ;* and a combination of other prophage-associated superantigen genes. No clear correlation was seen between clinical presentation and the presence of a particular profile of superantigens and/or DNases (Fig. S5).

### *emm*12

A total of 64/341 (18.8 %) 2022–2023 isolate genomes were *emm*12, the majority of which were throat isolates (52/64, 81.3 %), and 11 of these were associated with scarlet fever. Ten were skin swab samples, and two were ear swabs. For the 2016–2017 collection, 12 out of 124 (9.7 %) genomes were *emm*12, and all were from a throat source.

**Fig. 5.**
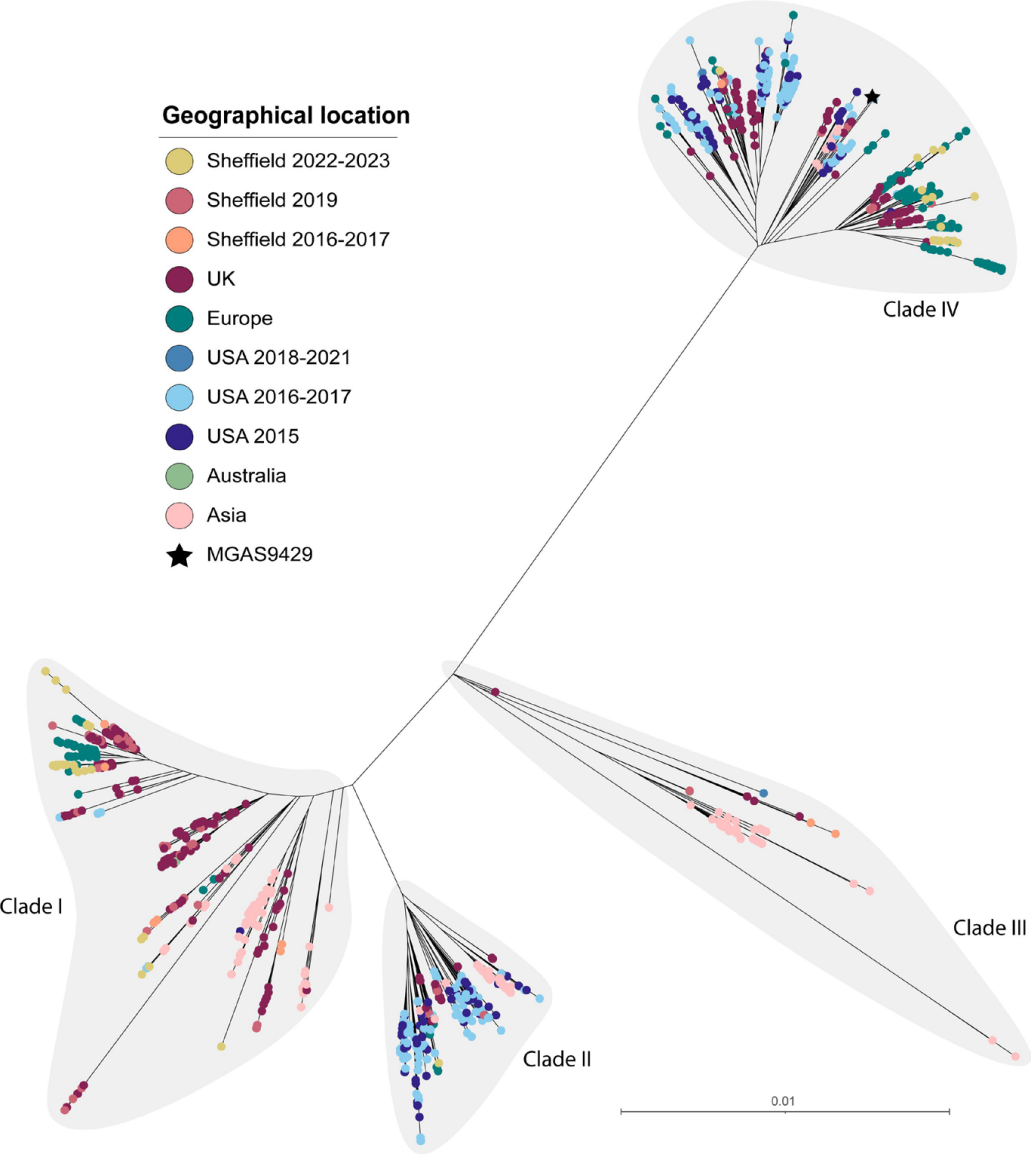
Phylogenetic analysis of Sheffield *emm*12 genomes collected in 2022–2023 and 2016–2017, within the context of global *emm*12 isolates. A maximum likelihood phylogenetic tree was generated with the core gene alignment (excluding prophage regions) to reference strain MGAS9429 [63]. Alongside our Sheffield *emm*12 genomes from 2022–2023 and 2016–2017, we included publicly available *emm*12 genome data from Sheffield, 2019 (*n*=15) [28]; the UK, 2009–2018 (*n*=439) [31,55, 56, 59]; Denmark, 2018–2023 (*n*=239) [14]; Portugal, 2022–2023 (*n*=12) [16]; the USA, 2015 (*n*=134) [38], 2016–2017 (*n*=228) [62] and 2018–2021 (*n*=2) [11]; Australia, 1995/2017 (*n*=2) [37,64]; Hong Kong, 2005–2011 (*n*=132) [37,65]; and China, 2004–2018 (*n*=43) [66]. Clades I–IV, previously defined by Davies *et al*. [37], are shaded grey. The scale bar represents the number of nucleotide substitutions per site.

The core genome phylogeny of global *emm*12 isolates showed four distinct clades (Fig. 5), as described previously [37]. The majority of all Sheffield isolates were clade I or clade IV, alongside other UK and European isolates. A single 2022–2023 Sheffield isolate was clade II, and no 2022–2023 isolates were found within clade III. Our phylogeny also showed three sub-clades within clade IV, one of which was dominated by Sheffield, other UK and European isolates, while isolates from the USA were restricted to the other two sub-clades, one of which also included Asian strains. Other US isolates also dominated clade II.

All Sheffield isolates were ST36, except four 2022–2023 isolates that were ST242 and clustered within one clade IV sub-clades (Fig. S6). One other 2022–2023 isolate was a single locus (*xpt*) variant ST1459. The only isolates to carry the *speA* gene were seven 2022–2023 throat isolates (9.1 %), and they clustered together within clade I. Again, no clear correlation was seen between clinical presentation and the presence of a particular profile of superantigens and/or DNases. Three 2022–2023 throat isolates in clade I carried *mefA* and *msrD* genes, associated with acquired macrolide resistance. Interestingly, ten 2022–2023 isolates with the CovR A105G variant all clustered together within clade I; a single isolate within this cluster also carried a RocA variation (G184R). A separate cluster of four isolates in clade IV carried the same mutation in *rocA* leading to a premature stop codon after 427 aa.

The single *emm*82 isolates from 2022–2023, and all seven *emm*82 isolates from 2016–2017 had the same ST as *emm*12: ST36. These isolates were confirmed to be the lineage of *emm*82 recently identified to have arisen through recombination and *emm*-switching of *emm*12 to *emm*82 [38,39].

### *emm*4

*emm*4 was associated with invasive disease in children <15 years early in the 2022–2023 upsurge [2] and was previously associated with the 2014 scarlet fever upsurge in England [4]. Of the twelve 2022–2023 *emm*4 isolates, nine were throat isolates, including three associated with scarlet fever; two were skin isolates, and one eye swab isolate. One skin-associated isolate had a highly divergent core genome and uniquely was ST289 and *emm-*subtype 4.2 rather than ST39 and *emm*-subtype 4.0 or 4.19. It also carried the *hasABC* locus which was characteristically absent in the other 11 *emm*4 isolates.

**Fig. 6.**
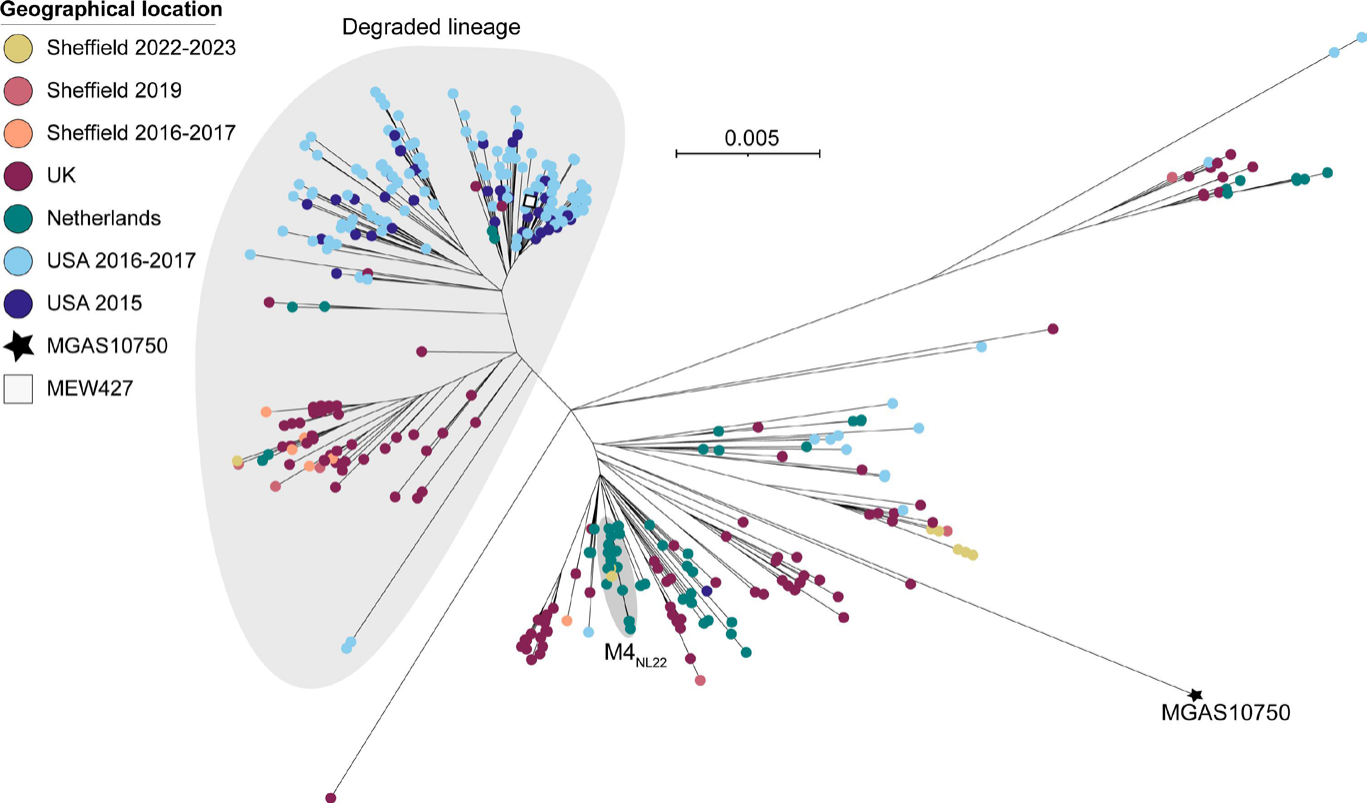
Phylogenetic analysis of Sheffield *emm*4 genomes collected in 2022–2023 and 2016–2017, within the context of global *emm*4 isolates. A maximum likelihood phylogenetic tree was generated with the core gene alignment (excluding prophage and SpyCI regions) to reference strain MGAS10750 (GenBank CP000262.1 [63]). The position of another reference strain MEW427 (GenBank CP014138.1 [67]) is also indicated. Alongside our Sheffield *emm*4 genomes from 2022–2023 and 2016–2017, we included publicly available *emm*4 genome data from Sheffield, 2019 (*n*=9) [28]; the UK, 2011–2016 (*n*=176) [31,55, 56, 59]; the Netherlands, 2009–2022 (*n*=66) [42]; and the USA, 2015–2017 (*n*=204) [38,62]. The large grey shaded area indicates the ‘Degraded’ lineage, and the small grey shaded area indicates the M4_NL22_ lineage. The scale bar represents the number of nucleotide substitutions per site.

Phylogeny of the other eleven 2022–2023 isolates within a wider *emm*4 population showed only a single strain clustering with the previously described ‘Degraded’ lineage [40] due to substantial loss of genes within the three prophages and the integrated conjugative element associated with *emm*4 (Fig. 6). This lineage also has a fusion of the 5′ of *emm* gene with the 3′ of the downstream *enn* gene [41]. The five *emm*4.19 isolates clustered together within a ‘Complete’ sub-lineage, closely related to other UK isolates. The remaining five isolates clustered with the recently described invasive disease-associated M4_NL22_ lineage from the Netherlands [42]. This appears to be a recent emergence of this lineage in England as no *emm*4 isolates in our 2016-2017 comparative collection nor in our Sheffield 2019 isolates [28] were found clustering with M4_NL22_ isolates. Our seven Sheffield 2016–2017 *emm*4 isolates included four within the ‘Degraded’ lineage, while three were ‘Complete’. This split was similar in our 2019 Sheffield isolates with four isolates in each of these lineages. This pattern of an even divide of UK isolates between ‘Complete’ and ‘Degraded’ lineages is consistent with our phylogeny and previous findings [40], with the shift towards dominance of the ‘Complete’ lineage, at least in Sheffield, emerging during the 2022–2023 upsurge.

### *emm*22

**Fig. 7.**
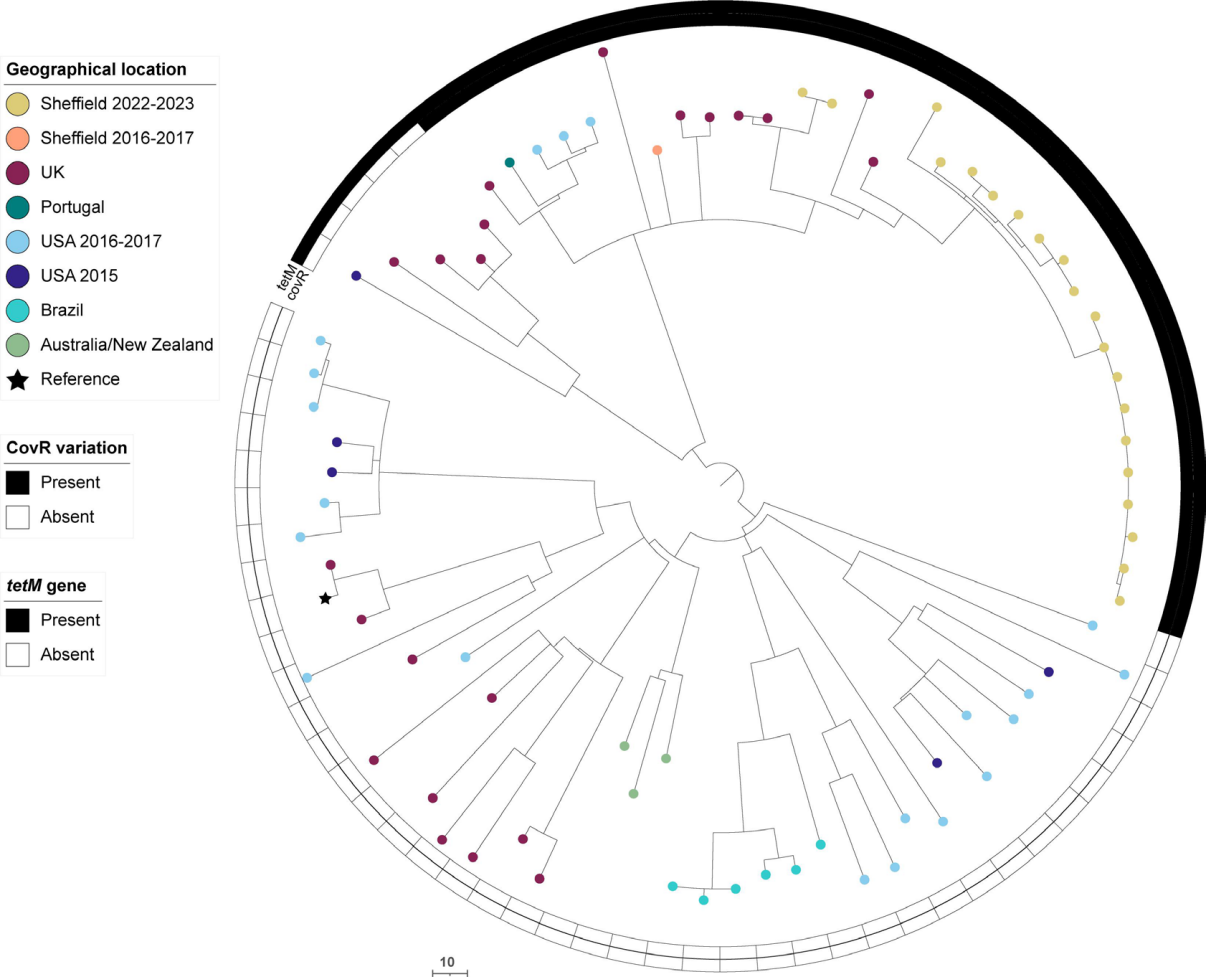
Phylogenetic analysis of Sheffield *emm*22 genomes collected in 2022–2023 and 2016–2017, within the context of global *emm*22 isolates. Alongside our Sheffield *emm*22 genomes from 2022–2023 and 2016–2017, we included publicly available *emm*22 genome data from the UK, 2001–2018 (*n*=22) [31,56]; Portugal, 2022–2023 (*n*=1) [16]; the USA, 2015 (*n*=5) [38] and 2016–2017 (*n*=20) [62]; Brazil, 2000–2013 (*n*=6) [68]; Australia, 1999/2004 (*n*=2) [68]; and New Zealand, 2010 (*n*=1) [68]. A maximum likelihood phylogenetic tree was generated with the core gene alignment to the *de novo* assembly of the oldest UK isolate, BSAC_bs1196 [31], and the removal of regions of potential recombination using Gubbins [26]. The scale bar represents the number of nucleotide substitutions per site.

A rise in *emm*22 was seen within the 2022–2023 collection, overall representing 5.9 % (20/341) of isolates compared with a single, skin-associated, isolate in 2016–2017. Of the 2022–2023 isolates, four were skin isolates and the remainder from throat, with 4/16 throat isolates also associated with scarlet fever. All were ST46. Phylogenetic analysis showed the clustering of all Sheffield 2022–2023 isolates, except two, together in expansion of a single lineage (Fig. 7). All Sheffield *emm*22 isolates carried a CovR V128A variant, clustering together in a lineage with 12 other isolates from the UK, Europe and the USA all also carrying the same variant. These isolates also carried *tetM*, alongside additional strains from the same parent lineage. All *emm*22 isolates, Sheffield and globally, carried the same variation in RocA (V333A) compared with a reference RocA sequence.

### *emm*75

**Fig. 8.**
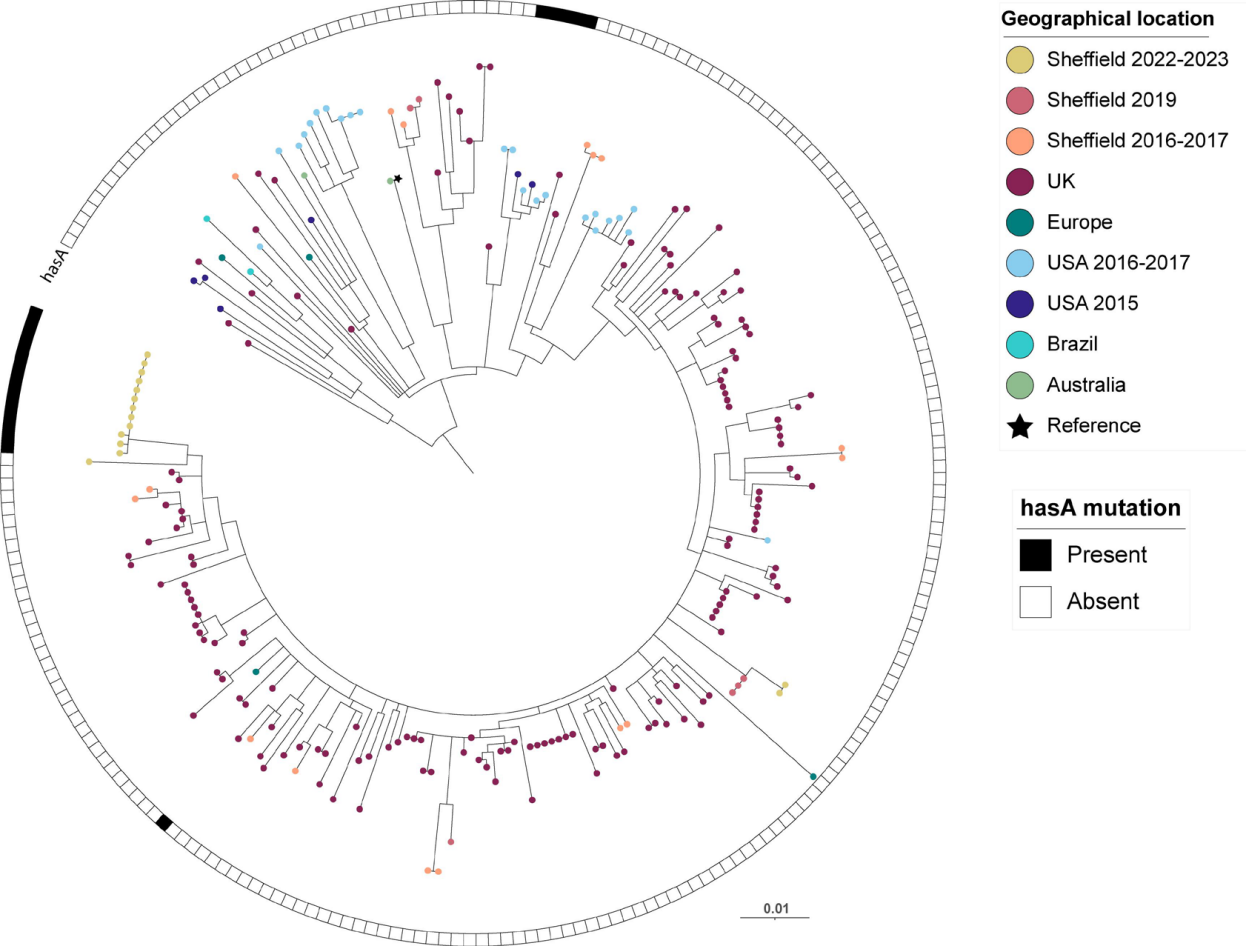
Phylogenetic analysis of Sheffield *emm*75 genomes collected in 2022–2023 and 2016–2017, within the context of global *emm*75 isolates. A maximum likelihood phylogenetic tree was generated with the core gene alignment to reference strain M75 (star) (GenBank CP033621.1 [69]). Alongside our Sheffield *emm*75 genomes from 2022–2023 and 2016–2017, we included publicly available *emm*75 genome data from Sheffield, 2019 (*n*=6) [28]; the UK, 2011–2016 (*n*=158) [31,55, 56, 59]; Belgium, 2004 (*n*=1) [68]; the USA, 2015–2017 (*n*=81) [38,62]; Australia, 1991–2010 (*n*=7) [68]; and Brazil, 2007–2012 (*n*=2) [68]. The scale bar represents the number of nucleotide substitutions per site. The presence or absence of a *hasA* nonsense mutation is indicated for each strain. Only isolates of ST150 (and closely related STs) are shown, excluding the other major lineage of *emm*75 (ST49 and closely related) as these are genetically quite distant [31]. Those that were ST49 and closely related STs comprised genomes from the UK, 2014–2018 (*n*=3) [56]; the USA, 2015 (*n*=14) and 2016–2017 (*n*=38) [38,62]; Australia, 1991–2004 (*n*=6) [68]; Fiji, 2006 (*n*=3) [68]; India, 2007–2010 (*n*=2) [68]; and Kenya, 2000–2011 (*n*=5) [68]. No Sheffield isolates were ST49.

Within all 2022–2023 Sheffield isolates, 4.4 % were *emm*75 (15/341), of which ten were from a throat source and four of these associated with scarlet fever, four from skin and one from an ear swab. By comparison, in 2016–2017, 9.1 % of isolates were *emm*75 (15/165), all of which were throat isolates not associated with scarlet fever. All Sheffield *emm*75 isolates from both 2022–2023 and 2016–2017 were ST150, distinct from the other dominant lineage of *emm*75, ST49. Of the 2022–2023 isolates, 13/15 formed a new sub-lineage with identical superantigen profiles (Fig. 8). Within this sub-lineage, 12/13 had the same additional T in a 7 residue homopolymeric tract in *hasA*, leading to a premature stop codon after 46 amino acids, and therefore are likely to be acapsular. This is new compared with 2016–2017 Sheffield isolates, none of which carried a *hasA* mutation and were scattered throughout the phylogeny. Other acapsular *emm*75 strains with the same premature stop codon in *hasA* were identified in a small cluster of US isolates, and a single sporadic UK strain.

### *emm*76, *emm*77 and *emm*87

Previously, we found recent expanding lineages within *emm*76, *emm*77 and *emm*87 that were acapsular due to mutations in *hasA* and had undergone recombination around the *nga-ifs-slo* promoter region to become high-toxin expressing variants [31]. In our 2022–2023 isolates, this was still the case for *emm*77 and *emm*87, but not for *emm*76.

In 2022–2023, 5.6 % (19/341) of isolates were *emm*76, compared with none in 2016–2017. Of these, 18 were from a skin source and one from an ear swab, and all were ST378, with no mutations in *hasA* or *hasB*, and predicted low-toxin expression (Fig. S7). All carried *tetM*, as described previously within this lineage [31]. This reflects a recent expansion of the encapsulated, low-toxin phenotype ST378 lineage, in contrast to the previously described ST50 lineage which was acapsular with high-toxin expression [31].

In 2022–2023, 4.1 % (14/341) of isolates were *emm*77, an increase from 2.4 % (4/165) in 2016–2017. Of the 2022–2023 isolates, 8 were from throat swabs and not associated with scarlet fever, 5 were from a skin source and 1 from an ear swab. The majority (11/14) were ST63, carrying resistance genes *tetO* and *ermA*, with the CovR M170I variation and belonged to the previously described acapsular (truncated HasA after 154 aa) with high-toxin expression lineage [31]. The two other *emm*77 isolates were ST399, quite distinct from ST63 although also predicted to be acapsular (truncated HasA after 46 aa) but with low-toxin expression.

An increase in *emm*87 was also observed in 2022–2023, 6.7 % (23/341), from 4.8 % in 2016–2017. Of the 2022–2023 isolates, 16 were from a throat source and 2 from ear swabs, 2 from eye swabs and 3 from skin sources. All isolates were part of the previously described lineage characterized as being acapsular, through a mutation in *hasA* (truncating HasA after 46 aa), and high-toxin expressing through recombination around the *nga-ifs-slo* locus and promoter [31] (Fig. S8). Interestingly, international phylogeny of *emm*87 revealed four broad clades, one of which contained a sub-lineage dominated by Sheffield and other UK strains and characterized by a premature stop codon in *hasB*, truncating it after 188 aa (Fig. S8). Only small clusters of USA *emm*87 isolates carried other truncating mutations in *hasB*. There was also some evidence of geographical variation across the lineages, with USA isolates dominating one lineage and UK isolates another.

### Invasive *S. pyogenes* isolates

During the time of our 2022–2023 collection, the Department of Laboratory Medicine, Sheffield, identified 19 iGAS samples (*S. pyogenes* isolated from a sterile site) and these had undergone routine *emm*-typing by the UKHSA, but genomic data was not available. Patients ranged in age from 1 to 94 years, but were most commonly 0–9 years (36.8 %, 7/19) or over 75 years (31.6 %, 6/19), in keeping with national data from the same time period. By far, the majority were *emm*1 (57.9 %, 11/19), followed by *emm*76 (15.8 %, 3/19), *emm*89 (10.5 %, 2/19), *emm*92 (10.5 %, 2/19) and *emm*87 (5.3 %, 1/19). The most common sample type was blood culture, at 13/19 samples (68.4 %, 3/19), followed by empyema samples (15.8 %, 3/19), and soft tissue or joint fluid (15.8 %, 3/19). All empyema samples were *emm*1 and all in children aged 0–9 years.

## Discussion

In recent years, the UK has experienced significant rises in morbidity and mortality in association with substantial upsurges in * S. pyogenes* infections, including in 2017–2018 and in 2022–2023 [3]. Despite this, study of non-invasive isolates has been limited, and the capacity for dynamic changes in non-invasive disease to drive upsurges and invasive disease is poorly understood. We sequenced 341 non-invasive isolates from Sheffield during the major 2022–2023 UK upsurge, and although we found *emm*1 and *emm*12 to be the leading causes of both throat and skin infections, they were differentially followed by *emm*22, *emm*87 and *emm*89 in throat infections but *emm*76 and *emm*49 in skin. A comparison to non-upsurge isolates from 2016–2017 indicated *emm*1 and *emm*12 contributed significantly to the 2022–2023 upsurge, but other *emm*-types had also changed over time with more *emm*22 in throat infections and *emm*76 in skin infections. All 2022–2023 *emm*1 isolates were the prevalent M1_UK_ lineage, but more diverse lineages were identified in other *emm*-types, including *emm*12, and emergent lineages in others, including *emm*75, demonstrating that, at least local to Sheffield, the upsurge was not primarily caused by a single genotype.

For both time periods studied, non-invasive *S. pyogenes* infections were most common in those aged 0–9 years, but more infections were seen in 5–9-year-old children in 2022–2023 than in 2016–2017 (33.3 % vs 15.7 %). Overall, those aged 4, 5, 6 or 7 years of age had the highest rates of *S. pyogenes* infection of any age in 2022–2023. This may reflect accelerated exposure to infection associated with school attendance, in keeping with reduced immunity to *S. pyogenes* and common respiratory viruses in this age group following reduced exposure during the COVID-19 pandemic [2,43].

The dominant peak in throat isolates at week 49 of 2022 within our collection, followed by a second smaller peak in week 3 of 2023, coincides with the peaks in scarlet fever notifications reported by the UKHSA during this time period [3]. During week 49, NHS England group A *Streptococcus* interim clinical guidance summary for case management was issued with guidance temporarily altering the clinical scoring criteria threshold for immediate antibiotic treatment in children with a sore throat. The issue of this guidance on the 9th of December 2022 likely enhanced clinician confidence in the empirical diagnosis of *S. pyogenes* throat infection and therefore may have contributed to a fall in throat swabs being received by the diagnostic laboratory. However, this also coincides with the fall in scarlet fever reporting nationally [3]. In contrast, the prominent peak in skin isolates occurred in week 52, between the peaks in throat isolates. This may be due to behavioural or environmental factors, such as increased skin-to-skin transmission of *S. pyogenes* associated with increased social mixing or altered chronic wound care at this time of year.

Analysis of national iGAS data from this time period identified a significant association between M1_UK_ and pleural isolates, likely the result of an early *S. pyogenes* upsurge coinciding with the respiratory virus season, facilitating disease progression [44]. Nationally, *emm*1 and *emm*12 were the most common *emm*-types among invasive isolates in all age groups during this upsurge, notably with *emm*4 as the next most common *emm*-type in children [2]. While we identified that 57.9 % of our invasive isolates were *emm*1, our dataset did not identify any invasive *emm*12 nor *emm*4 isolates, and the remaining 47.4 % (8/19) of invasive isolates during the 2022–2023 collection were of four other *emm*-types, potentially a reflection of regional variation in circulating strains within England. The skin prevalent *emm*76 did cause a substantial proportion of skin and iGAS infections suggesting we should not overlook this infection site. It is not known if the pronounced association of *emm*76 with skin infections was a local phenomenon as national data on skin infection *emm*-types were not collected.

Tissue tropism within *S. pyogenes* infections is well-recognized but bacterial molecular adaptations associated with tissue specialization remain incompletely understood [45]. An acapsular genotype was frequently seen in throat isolates in this study, with just 51 % predicted to be able to produce capsule compared with 78 % of skin isolates. In throat isolates, these acapsular strains were predominantly ‘generalist’ pattern E. Just 27 % of pattern E isolates overall were predicted to be encapsulated and made up 44.5 % of throat infections. Interestingly, although more skin than throat infections were pattern E, at 56 %, a higher proportion (62.5 %) of these were encapsulated. We also identified evidence of evolving capsule loss with the emergence of a recent acapsular sub-lineage of *emm*75, a pattern E type, and the increase in the prevalence of acapsular *emm*22.

We further observed tissue-specific differences in predicted expression of the toxins NADase and SLO based on the promoter sequence, with 90 % of throat isolates predicted to express high levels of toxins compared with 56 % of skin isolates, highlighting a key role for toxin expression in the pathogenesis of *S. pyogenes* throat infections. We previously provided evidence of convergent evolution with acapsular strains gaining increased toxin expression through homologous recombination [31]. Successful emergent lineages characterized by this recombination event have been identified previously in pattern E *emm*76, *emm*77 and *emm*87. Interestingly, while we continued to see expansion of these acapsular/high-toxin lineages in *emm*77 and *emm*87, in *emm*76, we observed instead an expansion of an ST378 encapsulated/low-toxin lineage to become a leading cause of skin infections. Indeed, overall, we observed significant differences between capsule/toxin-expression genotypes by infection site, with more throat isolates possessing an acapsular/high-toxin genotype and more skin isolates possessing an encapsulated/low-toxin genotype. This association was maintained even within the ‘generalist’ pattern E isolates when divided by isolate source, suggesting that differential capsule expression and NADase/SLO expression is a bacterial adaptation mechanism for tissue tropism. The emergence of lineages within pattern E *emm*-types 76, 77, 87 and 89 that have undergone genetic changes to become acapsular with high-toxin expression appear to be recent events [31]; the rise to dominance of the new acapsular/high-toxin variant of *emm*89 in the UK population occurred in 2007–2008 [33]. Here, in 2022–2023 compared with 2016–2017, we observed an increase in the prevalence of acapsular/high-toxin expression genotype *emm*22 causing throat infections and the emergence of an acapsular *emm*75 lineage. Why there has been a recent shift towards an acapsular/high toxin expression genotype and why this supports throat infections over skin infections is unclear. Although capsule has been shown to promote upper respiratory tract infections for some genotypes [46,47], long-term throat carriage isolates lose capsule expression through mutations in *hasABC* [48], potentially allowing for invasion into host cells that is otherwise hindered by capsule expression. Although why this would be preferential in the throat compared with the skin is also unclear. Capsule also provides protection against phagocytosis, and this has also been shown to promote both skin and throat infections [47]; however, this may be an *emm*-type specific phenomenon [49], and an increase in NADase/SLO expression may compensate for capsule loss [50]. An association between NADase activity and tissue tropism has also been identified previously, with NADase-inactive strains being primarily ‘skin-associated’ *emm*-pattern D, suggesting toxin may be less essential for *S. pyogenes* infection in the skin ecological niche compared with in the throat [51], although this could also be related to capsule expression in this niche, rendering NADase activity non-essential. We are limited in our study by the fact that we are only looking at genotypes and predicting phenotype, and it is possible that predicted capsule or NADase/SLO expression may be different *in vivo*. Work is ongoing to determine these phenotypes and the association with tissue tropism.

The most frequently identified *emm*-type across both throat and skin infections in 2022–2023 was *emm*1; all were M1_UK_ lineage and distributed throughout the M1_UK_ phylogeny without evidence of local expansion of a specific sub-clade. While classically ‘throat-associated’, it is likely the high number of *emm*1 cases in the skin reflected the high burden of throat disease and onwards transmission to the skin [52]. In contrast, *emm*12 isolates from our collections in both 2022–2023 and 2016–2017 were distributed predominantly across two of the four clades: I and IV. Previous work has suggested an association in clades I–III between scarlet fever and *ssa* (encoding streptococcal superantigen A), and an absence of scarlet fever in clade IV; however, no such clear associations were seen in our collection [37]. Geographical variation was apparent across our *emm*12 phylogeny, with the majority of Sheffield isolates found within a single sub-clade of clade IV and clade I, in a distribution distinct from that of USA isolates and again from Asian isolates. We observed similar geographical variation in *emm*87, with two of the four main clades dominated by USA strains and one clade containing the majority of Sheffield and other UK strains. These variations are in keeping with regional divergence of circulating lineages and can give rise to the emergence of geographically restricted sub-lineages, some of which have been seen to expand more widely if carrying a fitness advantage [7].

Comparison of isolates from 2022–2023 to 2016–2017 revealed differences in *emm*-type distribution between collections. More isolates from 2022–2023 were *emm*1 and *emm*12 compared with 2016–2017, across all infection sites. Within throat samples, 2022–2023 saw a fall in the number of *emm*89 and *emm*75 isolates compared with 2016–2017. Within skin site isolates, we saw more *emm*76 and *emm*49 in 2022–2023, and fewer *emm*43, *emm*89 and *emm*28 than 2016–2017. Where there is seasonality of *S. pyogenes* infection, as seen in Sheffield, seasonal fluctuation in *emm*-type distribution also frequently occurs. It is likely this is multifactorial, affected by antibiotic pressures, climate, patterns of social mixing and variations in concurrent circulating respiratory viruses. Population immunity also plays a key role in successful strain transmission; the strongest immunological protection is *emm*-type-specific, though infection with one *emm*-type can confer a degree of protection to other *emm*-types, thereby influencing the profile of circulating strains in subsequent seasons [53]. Genomic changes in circulating strains also have the potential to enhance strain virulence, with such changes underpinning previous upsurges in *S. pyogenes* disease [7]. We have demonstrated several emergent sub-lineages in our 2022–2023 isolates, including within *emm*22 and *emm*75, which is likely to further impact strain diversity between seasons.

Our study was limited by the lack of genomic data for local iGAS isolates; the addition of this data would allow a more detailed genomic comparison to better understand the interplay between non-invasive and invasive diseases. Our throat samples were deemed scarlet fever or not scarlet fever based on details provided by clinicians, which were often incomplete. Public health and media messaging may have also contributed to ascertainment bias. In weeks 49 and 50 of our study, our diagnostic laboratory received substantially more throat swabs compared with these same weeks in previous years, with high numbers of *S. pyogenes* identified, however, with a lower overall swab positivity.

Overall, we found that an increase in prevalence of *emm*1 and *emm*12 in non-invasive disease in 2022–2023 locally reflected the national increase of these *emm*-types in iGAS and scarlet fever cases. We have highlighted the need to study both throat and skin infection isolates as they can differ, and the potential for differential capsule expression and/or NADase and SLO toxin expression to drive tissue tropism. We also demonstrated that *emm*-types may not be represented by single lineages and these can change over time and by geographical location. Frequent monitoring with WGS is needed to determine how rapidly these changes occur, what factors influence these changes and how they might drive infection rates, including overspill from non-invasive disease into invasive and upsurges.

## supplementary material

10.1099/mgen.0.001277 Supplementary Material 1.

10.1099/mgen.0.001277 Supplementary Material 2.

10.1099/mgen.0.001277 Supplementary Material 3.

## Abbreviations

COVID-19: coronovirus disease 2019
iGAS: invasive *S. pyogenes*/group A *Streptococcus*
SLO: streptolysin O
StrepA: *Streptococcus pyogenes*
UKHSA: UK Health Security Agency
WGS: whole genome sequencing
