# Supplementary material for: Molecular characterization of Streptococcus pyogenes (StrepA) non-invasive isolates during the 2022–2023 UK upsurge
Source: Microb Genom. 2024 Aug 12;10(8):001277. doi: 10.1099/mgen.0.001277 (PMC11318961; doi:10.1099/mgen.0.001277)
Supplement: Supplementary Material 3. [file mgen-10-01277-s003.pdf]

Supplementary Information for:

Molecular characterisation of *Streptococcus pyogenes* (StrepA) non-invasive isolates during the 2022-23 UK upsurge.

Jennifer N. Hall[1,2,3,4], Saikou Y. Bah[2,3,4], Henna Khalid[2,3], Alison Brailey[5], Sarah Coleman[5], Tracey Kirk[5], Naveed Hussain[5], Mark Tovey[5], Roy R. Chaudhuri[2,3], Steve Davies[5], Lisa Tilley[5], Thushan de Silva[1,2], Claire E. Turner[2,3#]

1. Division of Clinical Medicine, School of Medicine and Population Health, University of Sheffield, Sheffield, United Kingdom
2. The Florey Institute of Infection, University of Sheffield, Sheffield, United Kingdom
3. School of Biosciences, University of Sheffield, Sheffield, United Kingdom
4. Medical Research Council Unit The Gambia at The London School of Hygiene & Tropical Medicine, Banjul, The Gambia
5. Laboratory Medicine, Sheffield Teaching Hospitals NHS Foundation Trust, Sheffield, United Kingdom

Supplementary Table 3: Project accession numbers for external sequence data used in study analyses.

Reference number	Reference	Project accession(s)
7	**Lynskey NN, Jauneikaite E, Li HK, Zhi X, Turner CE, *et al.*** Emergence of dominant toxigenic M1T1 *Streptococcus pyogenes* clone during increased scarlet fever activity in England: a population-based molecular epidemiological study. *Lancet Infect Dis* 2019;19:1209–1218. DOI: 10.1016/S1473-3099(19)30446-3	PRJEB12015
9	**Davies MR, Keller N, Brouwer S, Jespersen MG, Cork AJ, *et al.*** Detection of *Streptococcus pyogenes* M1UK in Australia and characterization of the mutation driving enhanced expression of superantigen SpeA. *Nat Commun* 2023;14:1051. DOI: 10.1038/s41467-023-36717-4.	PRJNA656382
11	**Li Y, Rivers J, Mathis S, Li Z, Chochua S, *et al.*** Expansion of Invasive Group A *Streptococcus* M1UK Lineage in Active Bacterial Core Surveillance, United States, 2019–2021. *Emerg Infect Dis* 2023;29:2116-2120. DOI: 10.3201/eid2910.230675.	PRJNA395240
13	**Rümke LW, de Gier B, Vestjens SMT, van der Ende A, van Sorge, *et al.*** Dominance of M1UK clade among Dutch M1 Streptococcus pyogenes. *Lancet Infect Dis* 2020;20:539-540. DOI: 10.1016/S1473-3099(20)30278-4.	PRJEB38751
14	**Johannesen TB, Munkstrup C, Edslev SM, Baig S, Nielsen S, *et al.*** Increase in invasive group A streptococcal infections and emergence of novel, rapidly expanding sub-lineage of the virulent *Streptococcus pyogenes* M1 clone, Denmark, 2023. *Eurosurveillance* 2023;28:2300291. DOI: 10.2807/1560-7917.ES.2023.28.26.2300291.	PRJEB62635, PRJEB62579, PRJEB62874
16	**Gouveia C, Bajanca-Lavado MP, Mamede R, Carvalho AA, Rodrigues F, *et al.*** Sustained increase of paediatric invasive *Streptococcus pyogenes* infections dominated by M1UK and diverse *emm*12 isolates, Portugal, September 2022 to May 2023. *Eurosurveillance* 2023;28:2300427. DOI: 10.2807/1560-7917.ES.2023.28.36.2300427	PRJEB65018
28	**Bah SY, Keeley AJ, Armitage EP, Khalid H, Chaudhuri RR, *et al.*** Genomic Characterization of Skin and Soft Tissue Streptococcus pyogenes Isolates from a Low-Income and a High-Income Setting. *mSphere* 2023;8:e0046922. DOI: 10.1128/msphere.00469-22.	PRJNA730523

31	**Turner CE, Holden MTG, Blane B, Horner C, Peacock SJ, *et al.*** The emergence of successful *Streptococcus pyogenes* lineages through convergent pathways of capsule loss and recombination directing high toxin expression. *mBio* 2019;10:e02521-19. DOI: 10.1128/mbio.02521-19.	PRJEB4679, PRJNA395240
38	**Chalker V, Jironkin A, Coelho J, Al-Shahib A, Platt S, *et al.*** Genome analysis following a national increase in Scarlet Fever in England 2014. *BMC Genomics* 2017;18:1–10. DOI: 10.1186/s12864-017-3603-z	PRJEB13551
39	**Kapatai G, Coelho J, Platt S, Chalker VJ**. Whole genome sequencing of group A *Streptococcus*: development and evaluation of an automated pipeline for *emm* gene typing. *PeerJ* 2017;5:e3226. DOI: 10.7717/peerj.3226	PRJEB17673
40	**Cordery R, Purba AK, Begum L, Mills E, Mosavie M, *et al.*** Frequency of transmission, asymptomatic shedding, and airborne spread of *Streptococcus pyogenes* in schoolchildren exposed to scarlet fever: a prospective, longitudinal, multicohort, molecular epidemiological, contact-tracing study in England, UK. *Lancet Microbe* 2022;3:e366–e375. DOI: 10.1016/S2666-5247(21)00332-3	PRJEB43915
41	**Sharma H, Ong MR, Ready D, Coelho J, Groves N, *et al.*** Real-time whole genome sequencing to control a *Streptococcus pyogenes* outbreak at a national orthopaedic hospital. *J Hosp Infect* 2019;103:21–26. DOI: 10.1016/j.jhin.2019.07.003	PRJEB29459
42	**Turner CE, Bedford L, Brown NM, Judge K, Török ME, *et al.*** Community outbreaks of group A *Streptococcus* revealed by genome sequencing. *Sci Rep* 2017;7:8554. DOI: 10.1038/s41598-017-08914-x	PRJEB4679
43	**Chen M, Cai J, Davies MR, Li Y, Zhang C, *et al.*** Increase of *emm*1 isolates among group A *Streptococcus* strains causing scarlet fever in Shanghai, China. *Int J Infect Dis* 2020;98:305–314. DOI: 10.1016/j.ijid.2020.06.053	PRJEB35406
44	**Ben Zakour NL, Davies MR, You Y, Chen JHK, Forde BM, *et al.*** Transfer of scarlet fever-associated elements into the group A *Streptococcus* M1T1 clone. *Sci Rep* 2015;5:15877. DOI: 10.1038/srep15877	PRJEB2839
45	**Chochua S, Metcalf BJ, Li Z, Rivers J, Mathis S, *et al.*** Population and Whole Genome Sequence Based Characterization of Invasive Group A Streptococci Recovered in the United States during 2015. *mBio* 2017;8:e01422-17. DOI: 10.1128/mbio.01422-17.	PRJNA395240
46	**Li Y, Rivers J, Velusamy S, McGee L**. Genomic Surveillance of *Streptococcus pyogenes* Strains Causing Invasive Disease, United States, 2016–2017. *Front Microbiol* 2020;11:1547. DOI: 10.3389/fmicb.2020.01547.	PRJNA395240

47	**Davies MR, Holden MT, Coupland P, Chen JHK, Venturini C, *et al.*** Emergence of scarlet fever *Streptococcus pyogenes emm*12 clones in Hong Kong is associated with toxin acquisition and multidrug resistance. *Nat Genet* 2015;47:84–87. DOI: 10.1038/ng.3147	PRJEB2657
51	**Tse H, Bao JYJ, Davies MR, Maamary P, Tsoi H-W, *et al.*** Molecular Characterization of the 2011 Hong Kong Scarlet Fever Outbreak. *J Infect Dis* 2012;206:341–351. DOI: 10.1093/infdis/jis362	PRJNA233611
52	**You Y, Davies MR, Protani M, McIntyre L, Walker MJ, *et al.*** Scarlet Fever Epidemic in China Caused by *Streptococcus pyogenes* Serotype M12: Epidemiologic and Molecular Analysis. *eBioMedicine* 2018;28:128–135. DOI: 10.1016/j.ebiom.2018.01.010	PRJNA416675
55	**Putten BCL van der, Bril-Keijzers WCM, Rumke LW, Vestjens SMT, Koster LAM, *et al.*** Novel *emm*4 lineage associated with an upsurge in invasive group A streptococcal disease in the Netherlands, 2022. *Microb Genomics* 2023;9:mgen001026. DOI: 10.1099/mgen.0.001026.	PRJEB58654
57	**Davies MR, McIntyre L, Mutreja A, Lacey JA, Lees JA, *et al.*** Atlas of group A streptococcal vaccine candidates compiled using large-scale comparative genomics. *Nat Genet* 2019;51:1035–1043. DOI: 10.1038/s41588-019-0417-8	PRJEB2232
63	**Athey TBT, Teatero S, Li A, Marchand-Austin A, Beall BW, Fittipaldi N.** Deriving group A Streptococcus typing information from short-read whole-genome sequencing data. *J Clin Microbiol* 2014;52:1871-6. DOI: 10.1128/JCM.00029-14	PRJNA233611

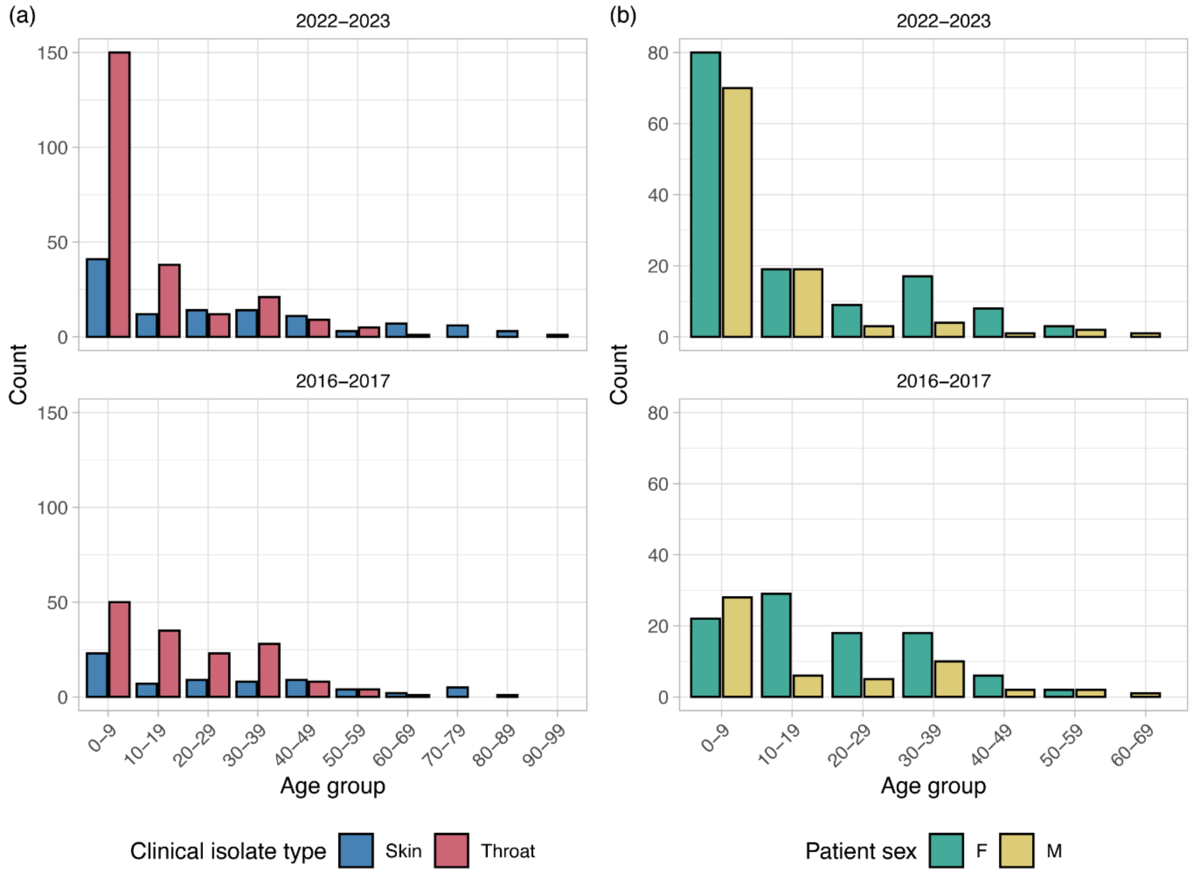

Supplementary Figure 1: (a) Age distribution of patients from whom isolates were collected in 2022-2023 and 2016-2017, grouped by clinical isolate type. Other clinical isolate types (eye, ear and nose) are excluded. (b) Age distribution of patients across throat isolates in 2022-2023 and 2016-2017, grouped by patient sex. No throat isolates were received from individuals aged 70 or older.

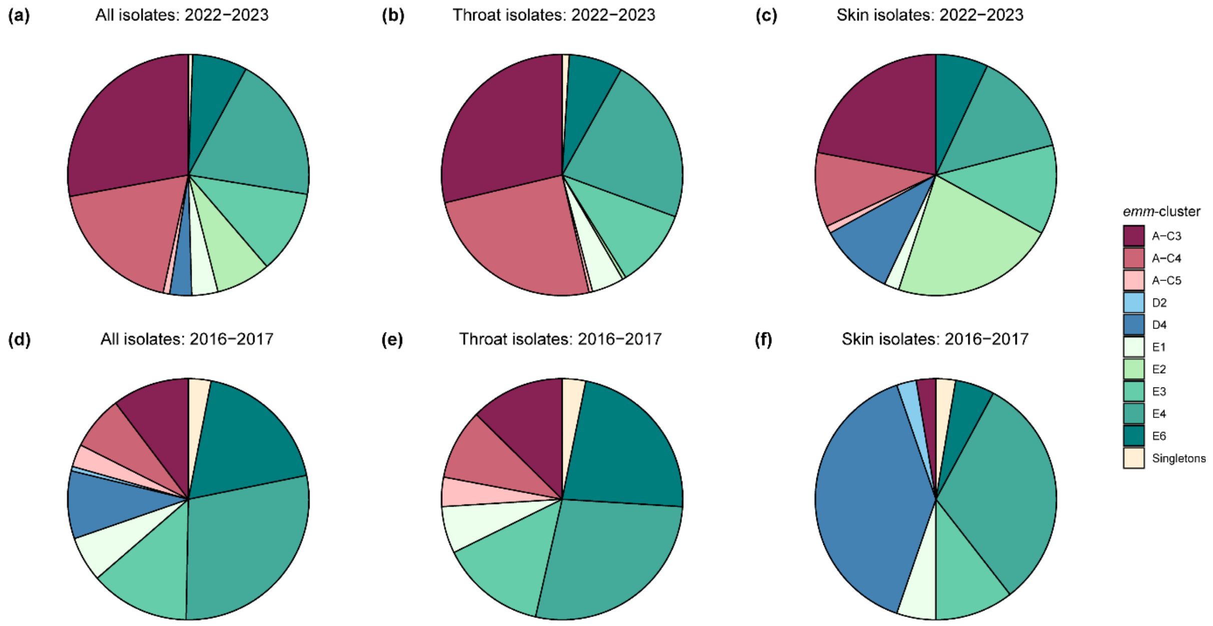

Supplementary Figure 2: **Distribution of *emm*-clusters across 2022-2023 and 2016-2017 collections**. (a) All 2022-2023 isolates; (b) 2022-2023 throat isolates; (c) 2022-2023 skin isolates; (d) all 2016-2017 isolates; (e) 2016-2017 throat isolates; (f) 2016-2017 skin isolates. Pie charts represent the percentage of isolates associated with each cluster.

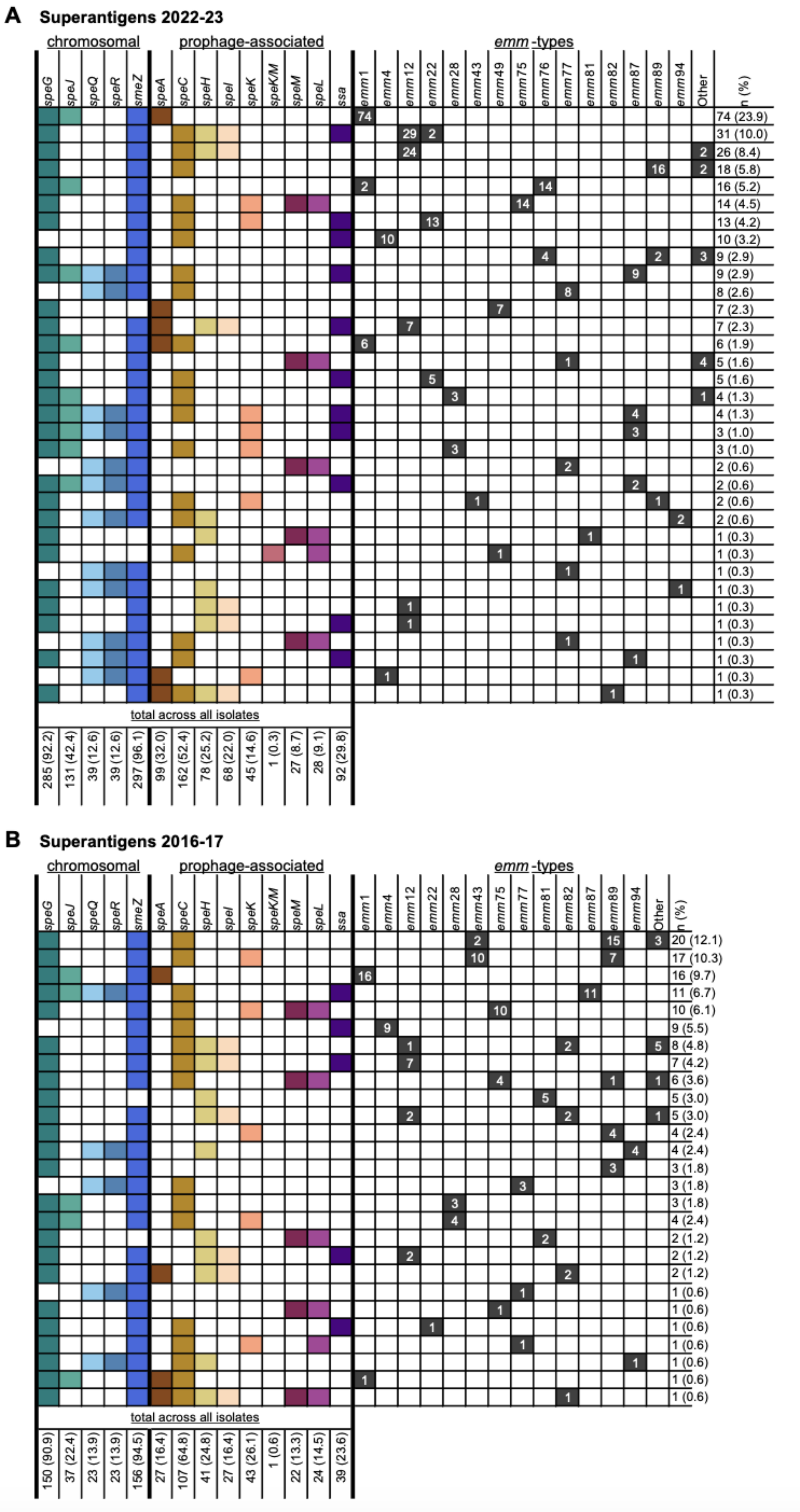
A Superantigens 2022-23
chromosomal
prophage-associated
emm-types
speG speJ speQ speR smeZ
speA speC speH speI speK speK/M speM speL ssa
emm1 emm4 emm12 emm22 emm28 emm43 emm49 emm75 emm76 emm77 emm81 emm82 emm87 emm89 emm94 Other
n (%)
74 (23.9)
31 (10.0)
26 (8.4)
18 (5.8)
16 (5.2)
14 (4.5)
13 (4.2)
10 (3.2)
9 (2.9)
9 (2.9)
8 (2.6)
7 (2.3)
7 (2.3)
6 (1.9)
5 (1.6)
5 (1.6)
4 (1.3)
4 (1.3)
3 (1.0)
3 (1.0)
2 (0.6)
2 (0.6)
2 (0.6)
2 (0.6)
1 (0.3)
1 (0.3)
1 (0.3)
1 (0.3)
1 (0.3)
1 (0.3)
1 (0.3)
1 (0.3)
1 (0.3)
1 (0.3)
total across all isolates
285 (92.2)
131 (42.4)
39 (12.6)
39 (12.6)
297 (96.1)
99 (32.0)
162 (52.4)
78 (25.2)
68 (22.0)
45 (14.6)
1 (0.3)
27 (8.7)
28 (9.1)
92 (29.8)
B Superantigens 2016-17
chromosomal
prophage-associated
emm-types
speG speJ speQ speR smeZ
speA speC speH speI speK speK/M speM speL ssa
emm1 emm4 emm12 emm22 emm28 emm43 emm75 emm77 emm81 emm82 emm87 emm89 emm94 Other
n (%)
20 (12.1)
17 (10.3)
16 (9.7)
11 (6.7)
10 (6.1)
9 (5.5)
8 (4.8)
7 (4.2)
6 (3.6)
5 (3.0)
5 (3.0)
4 (2.4)
4 (2.4)
3 (1.8)
3 (1.8)
3 (1.8)
4 (2.4)
2 (1.2)
2 (1.2)
2 (1.2)
1 (0.6)
1 (0.6)
1 (0.6)
1 (0.6)
1 (0.6)
1 (0.6)
1 (0.6)
total across all isolates
150 (90.9)
37 (22.4)
23 (13.9)
23 (13.9)
156 (94.5)
27 (16.4)
107 (64.8)
41 (24.8)
27 (16.4)
43 (26.1)
1 (0.6)
22 (13.3)
24 (14.5)
39 (23.6)

Supplementary Figure 3: Summary of superantigen profiles by *emm*-type in throat and skin isolates collected in (a) 2022-2023 and (b) 2016-2017. Data shown for the most common *emm*-types ordered from most common profile to least common; other *emm*-types are categorised here as 'Other'. Superantigen combinations found only in 'Other' *emm*-types have been excluded. Individual superantigen totals at the bottom of the figure are displayed as n (%) and represent all skin and throat isolates of all *emm*-types, including those only found in 'other' *emm*-types.

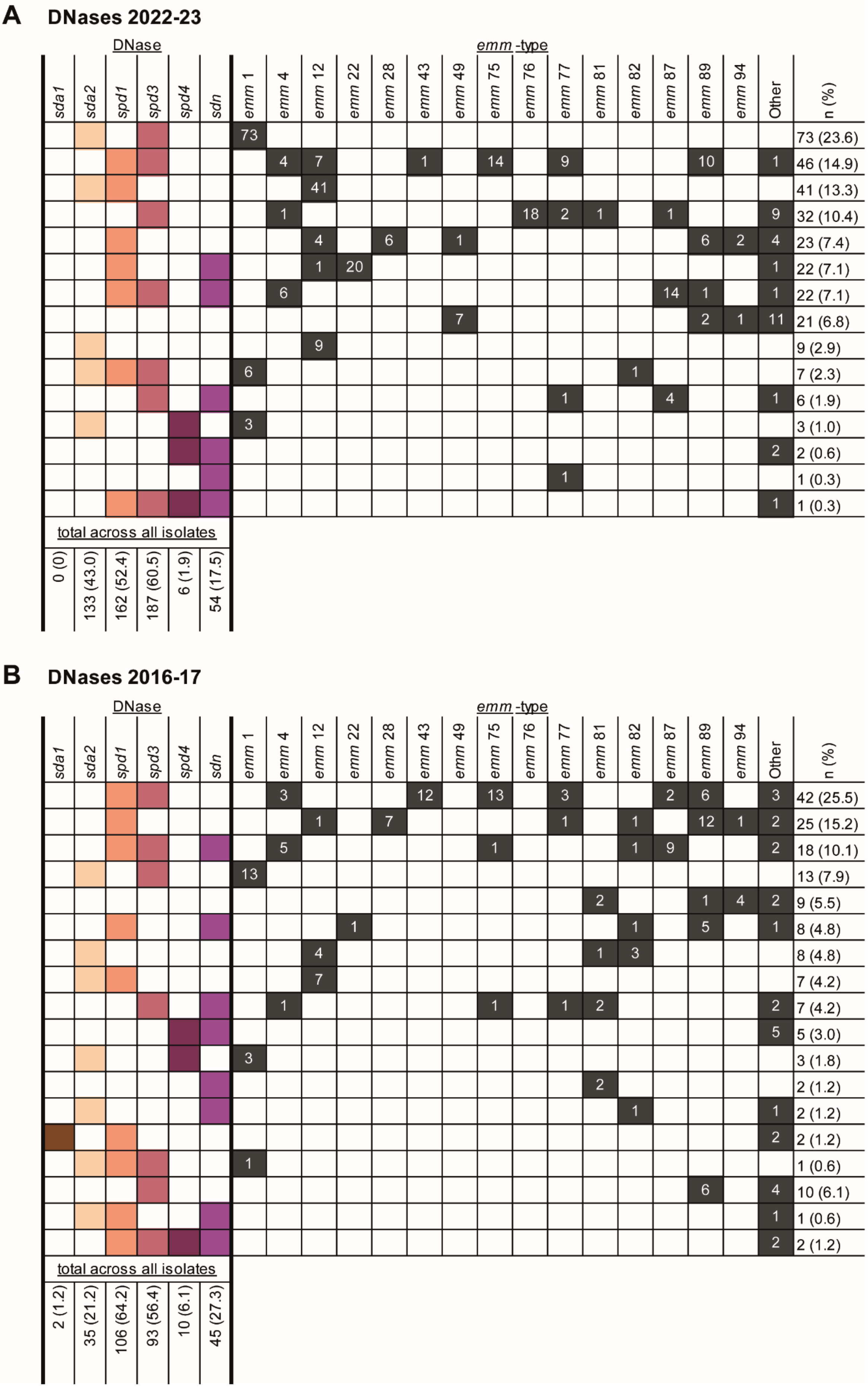
A
DNases 2022-23
DNase
sda1
sda2
spd1
spd3
spd4
sdn
emm -type
emm 1
emm 4
emm 12
emm 22
emm 28
emm 43
emm 49
emm 75
emm 76
emm 77
emm 81
emm 82
emm 87
emm 89
emm 94
Other
n (%)
73
73 (23.6)
4 7 1 14 9 10 1
46 (14.9)
41
41 (13.3)
1 18 2 1 1 9
32 (10.4)
4 6 1 6 2 4
23 (7.4)
1 20 1
22 (7.1)
6 14 1 1
22 (7.1)
7 2 1 11
21 (6.8)
9
9 (2.9)
6 1
7 (2.3)
1 4 1
6 (1.9)
3
3 (1.0)
2
2 (0.6)
1
1 (0.3)
1
1 (0.3)
total across all isolates
0 (0)
133 (43.0)
162 (52.4)
187 (60.5)
6 (1.9)
54 (17.5)
B
DNases 2016-17
DNase
sda1
sda2
spd1
spd3
spd4
sdn
emm -type
emm 1
emm 4
emm 12
emm 22
emm 28
emm 43
emm 49
emm 75
emm 76
emm 77
emm 81
emm 82
emm 87
emm 89
emm 94
Other
n (%)
3 12 13 3 2 6 3
42 (25.5)
1 7 1 1 12 1 2
25 (15.2)
5 1 1 9 2
18 (10.1)
13
13 (7.9)
2 1 4 2
9 (5.5)
1 1 5 1
8 (4.8)
4 1 3
8 (4.8)
7
7 (4.2)
1 1 1 2 2
7 (4.2)
5
5 (3.0)
3
3 (1.8)
2
2 (1.2)
1 1
2 (1.2)
2
2 (1.2)
1
1 (0.6)
6 4
10 (6.1)
1
1 (0.6)
2
2 (1.2)
total across all isolates
2 (1.2)
35 (21.2)
106 (64.2)
93 (56.4)
10 (6.1)
45 (27.3)

Supplementary Figure 4: Summary of DNase profiles by *emm*-type in throat and skin isolates collected in (a) 2022-2023 and (b) 2016-2017. Data shown for the most common *emm*-types ordered from most common profile to least common; other *emm*-types are categorised here as ‘Other’. Individual DNase totals at the bottom of the figure are displayed as n (%) and represent all skin and throat isolates of all *emm*-types.

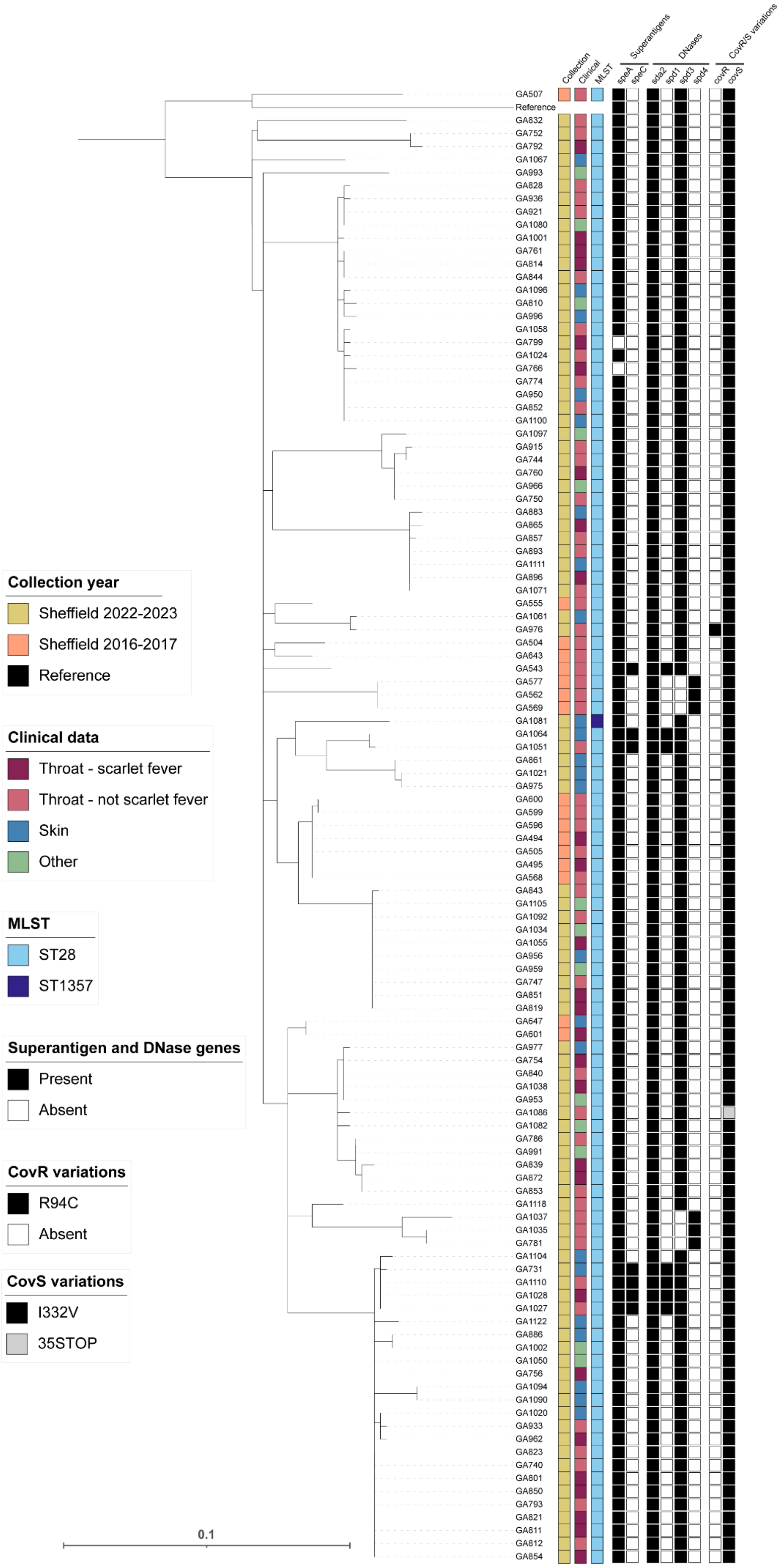

Collection
Clinical
MLST
Superantigens
speA
speC
DNases
sda2
spd1
spd3
spd4
CovR/S variations
covR
covS
GA507
Reference
GA832
GA752
GA792
GA1067
GA993
GA828
GA936
GA921
GA1080
GA1001
GA761
GA814
GA844
GA1096
GA810
GA996
GA1058
GA799
GA1024
GA766
GA774
GA950
GA852
GA1100
GA1097
GA915
GA744
GA760
GA966
GA750
GA883
GA865
GA857
GA893
GA1111
GA896
GA1071
GA555
GA1061
GA976
GA504
GA643
GA543
GA577
GA562
GA569
GA1081
GA1064
GA1051
GA861
GA1021
GA975
GA600
GA599
GA596
GA494
GA505
GA495
GA568
GA843
GA1105
GA1092
GA1034
GA1055
GA956
GA959
GA747
GA851
GA819
GA647
GA601
GA977
GA754
GA840
GA1038
GA953
GA1086
GA1082
GA786
GA991
GA839
GA872
GA853
GA1118
GA1037
GA1035
GA781
GA1104
GA731
GA1110
GA1028
GA1027
GA1122
GA886
GA1002
GA1050
GA756
GA1094
GA1090
GA1020
GA933
GA962
GA823
GA740
GA801
GA850
GA793
GA821
GA811
GA812
GA854
Collection year
Sheffield 2022-2023
Sheffield 2016-2017
Reference
Clinical data
Throat - scarlet fever
Throat - not scarlet fever
Skin
Other
MLST
ST28
ST1357
Superantigen and DNase genes
Present
Absent
CovR variations
R94C
Absent
CovS variations
I332V
35STOP
0.1

Supplementary Figure 5: **Phylogenetic analysis of the 112 Sheffield *emm*1 genomes collected in 2022-2023 and 2016-2017.** A maximum likelihood phylogenetic tree was generated with the core gene alignment to reference strain MGAS5005. The scale bar represents the number of nucleotide substitutions per site. Colour strips indicate the year of isolate collection, clinical presentation and the multi-locus sequence type (MLST). The presence (black) and absence (white) of superantigen genes *speA* and *speC*; prophage-associated DNase genes *sda2*, *spd1*, *spd3*, and *spd4* are indicated. Variations in CovR and CovS are indicated. All isolates possessed chromosomal superantigens *speG*, *speJ* and *smeZ*. No isolates had superantigen genes *speH*, *speI*, *speK*, *speL*, *speK/M*, *speM*, *speQ*, *speR* or *ssa*, nor DNase genes *sda1* or *sdn*. No antimicrobial resistance (AMR) genes were identified within the Sheffield *emm*1 isolates. All Sheffield *emm*1 isolates also had a Q259R variation in RocA.

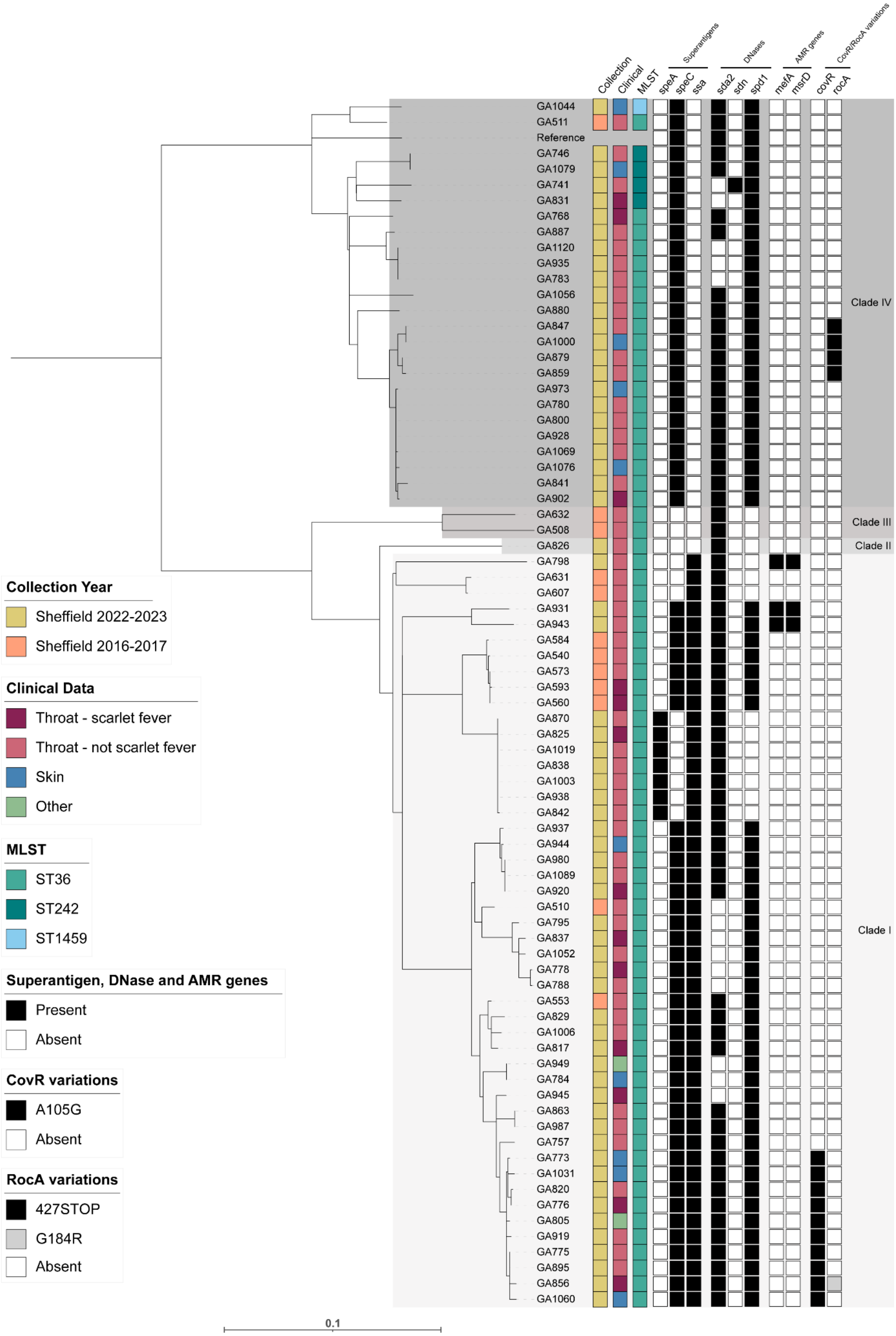
Collection
Clinical
MLST
Superantigens
speA
speC
ssa
DNases
sda2
sdn
spd1
AMR genes
mefA
msrD
CovR/RocA variations
covR
rocA
Clade IV
Clade III
Clade II
Clade I
Reference
0.1
Collection Year
Sheffield 2022-2023
Sheffield 2016-2017
Clinical Data
Throat - scarlet fever
Throat - not scarlet fever
Skin
Other
MLST
ST36
ST242
ST1459
Superantigen, DNase and AMR genes
Present
Absent
CovR variations
A105G
Absent
RocA variations
427STOP
G184R
Absent

Supplementary Figure 6: **Phylogenetic analysis of the 77 Sheffield *emm*12 genomes collected in 2022-2023 and 2016-2017.** A maximum likelihood phylogenetic tree was generated with the core gene alignment to reference strain MGAS9429. The scale bar represents the number of nucleotide substitutions per site. Colour strips indicate the year of isolate collection and the multi-locus sequence type (MLST). The presence (grey) and absence (white) of superantigen genes *speA*, *speC* and *ssa*; DNase genes *sda2*, *sdn, spd1* and *spd3*; and variations in CovR and RocA are indicated. No variations in CovS were identified within Sheffield *emm*12 genomes. All isolates possessed DNase genes *speG, speH* and *speI*. No isolates had superantigens *speJ, speK, speK/M, speM, speL, speQ, speR* or *smeZ*, nor DNase genes *sda1* and *spd4*. The presence (grey) and absence (white) of antimicrobial resistance (AMR) genes *mefA* and *msrD* is indicated; no other AMR genes were identified within Sheffield *emm*12 isolates.

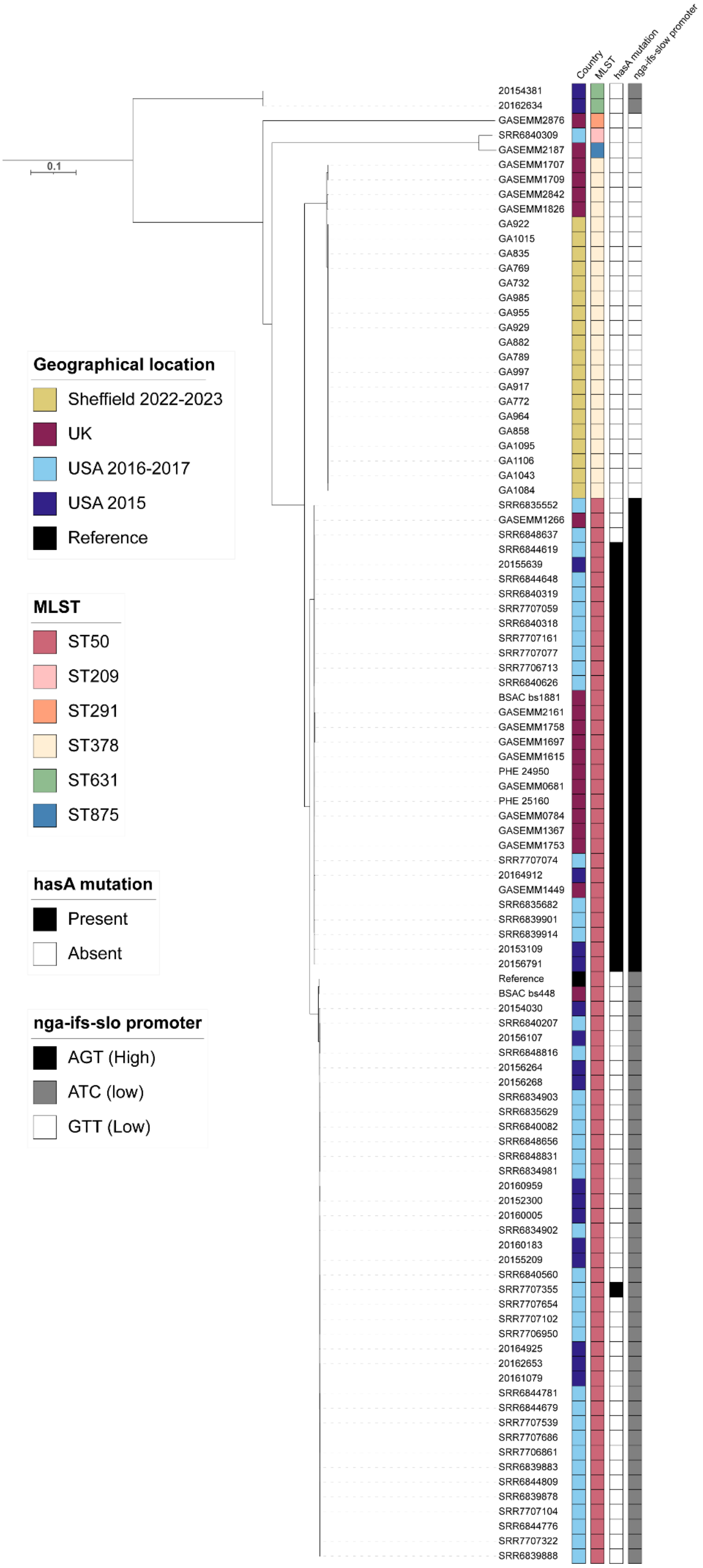
Country
MLST
hasA mutation
nga-ifs-slow promoter
0.1
20154381
20162634
GASEMM2876
SRR6840309
GASEMM2187
GASEMM1707
GASEMM1709
GASEMM2842
GASEMM1826
GA922
GA1015
GA835
GA769
GA732
GA985
GA955
GA929
GA882
GA789
GA997
GA917
GA772
GA964
GA858
GA1095
GA1106
GA1043
GA1084
SRR6835552
GASEMM1266
SRR6848637
SRR6844619
20155639
SRR6844648
SRR6840319
SRR7707059
SRR6840318
SRR7707161
SRR7707077
SRR7706713
SRR6840626
BSAC bs1881
GASEMM2161
GASEMM1758
GASEMM1697
GASEMM1615
PHE 24950
GASEMM0681
PHE 25160
GASEMM0784
GASEMM1367
GASEMM1753
SRR7707074
20164912
GASEMM1449
SRR6835682
SRR6839901
SRR6839914
20153109
20156791
Reference
BSAC bs448
20154030
SRR6840207
20156107
SRR6848816
20156264
20156268
SRR6834903
SRR6835629
SRR6840082
SRR6848656
SRR6848831
SRR6834981
20160959
20152300
20160005
SRR6834902
20160183
20155209
SRR6840560
SRR7707355
SRR7707654
SRR7707102
SRR7706950
20164925
20162653
20161079
SRR6844781
SRR6844679
SRR7707539
SRR7707686
SRR7706861
SRR6839883
SRR6844809
SRR6839878
SRR7707104
SRR6844776
SRR7707322
SRR6839888
Geographical location
Sheffield 2022-2023
UK
USA 2016-2017
USA 2015
Reference
MLST
ST50
ST209
ST291
ST378
ST631
ST875
hasA mutation
Present
Absent
nga-ifs-slo promoter
AGT (High)
ATC (low)
GTT (Low)

Supplementary Figure 7: **Phylogenetic analysis of Sheffield *emm*76 genomes collected in 2022-2023, within the context of other UK and USA *emm*76 isolates.** Alongside our Sheffield *emm*76 genomes from 2022-2023 and 2016-2017 we included publicly available *emm*76 genome data from the UK, 2002-2018 (n=20) (31,38,39); USA, 2015 (n=18) (45) and 2016-2017 (n=42) (46). A maximum likelihood phylogenetic tree was generated with the core gene alignment to reference strain BSAC_bs448. The scale bar represents the number of nucleotide substitutions per site. Colour strips indicate the country of isolate collection and the multi-locus sequence type (MLST). The presence (black) and absence (white) of *hasA* mutations is indicated, as are variants of the *nga-ifs-slo* promoter.

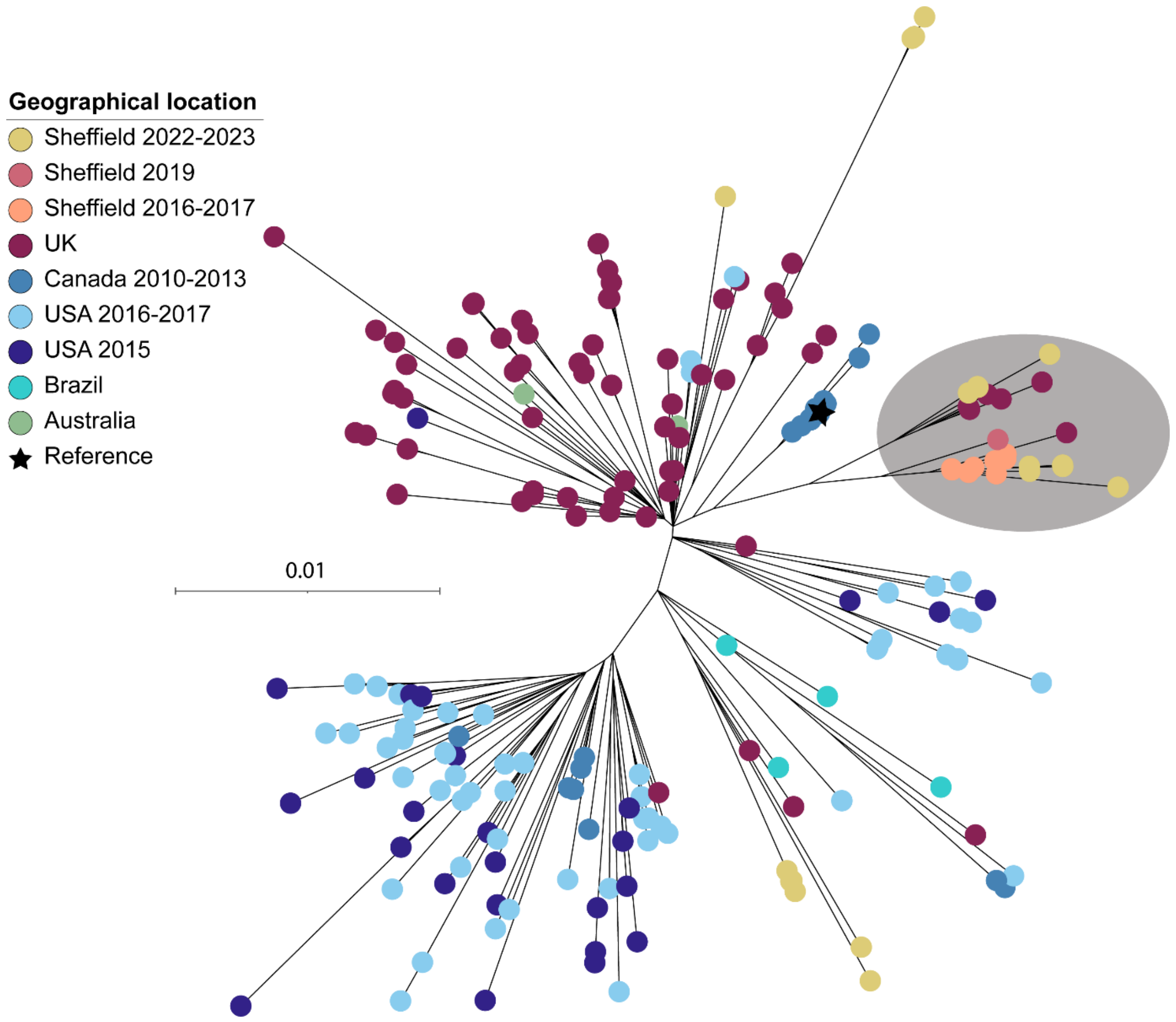

Supplementary Figure 8: **Phylogenetic analysis of Sheffield *emm*87 genomes collected in 2022-2023 and 2016-2017, within the context of global *emm*87 isolates.** Alongside our Sheffield *emm*87 genomes from 2022-2023 and 2016-2017 we included publicly available *emm*87 genome data from the UK, 2001-2018 (n=91) (31,38,39); USA, 2015 (n=26) (45); USA, 2016-2017 (n=40) (46); Canada, 2010-2013 (n=22) (70); Brazil, 2000-2013 (n=4) (57); and New Zealand, 2009-2010 (n=2) (57). A maximum likelihood phylogenetic tree was generated with the core gene alignment to reference strain NGAS743 (star). All isolates have a mutation in *hasA* that would truncate HasA but isolates in the lineage shaded in grey have an additional mutation in *hasB* that would truncate HasB. The

scale bar represents the number of nucleotide substitutions per site. Two highly divergent UK strains were excluded from the tree for presentation purposes.

Supplementary References

70. **Athey TBT, Teatero S, Li A, Marchand-Austin A, Beall BW, *et al*.** Deriving group A *Streptococcus* typing information from short-read whole-genome sequencing data. *J Clin Microbiol* 2014;52:1871–1876.
